# Manipulating the Coordination Structure of Molecular Cobalt Sites in Periodic Mesoporous Organosilica for CO_2_ Photoreduction

**DOI:** 10.1021/acsaem.4c01161

**Published:** 2024-06-29

**Authors:** Raúl Rojas-Luna, Francisco J. Romero-Salguero, Dolores Esquivel, Souvik Roy

**Affiliations:** † Departamento de Química Orgánica, Instituto Químico para la Energía y el Medioambiente (IQUEMA), Facultad de Ciencias, Universidad de Córdoba, Campus de Rabanales, Edificio Marie Curie, Córdoba E-14071, Spain; ‡ School of Chemistry, University of Lincoln, Green Lane, Lincoln LN6 7DL, U.K.

**Keywords:** periodic mesoporous organosilica, CO_2_ photoreduction, single cobalt sites, coordination structure, heterogenization, polypyridine ligands

## Abstract

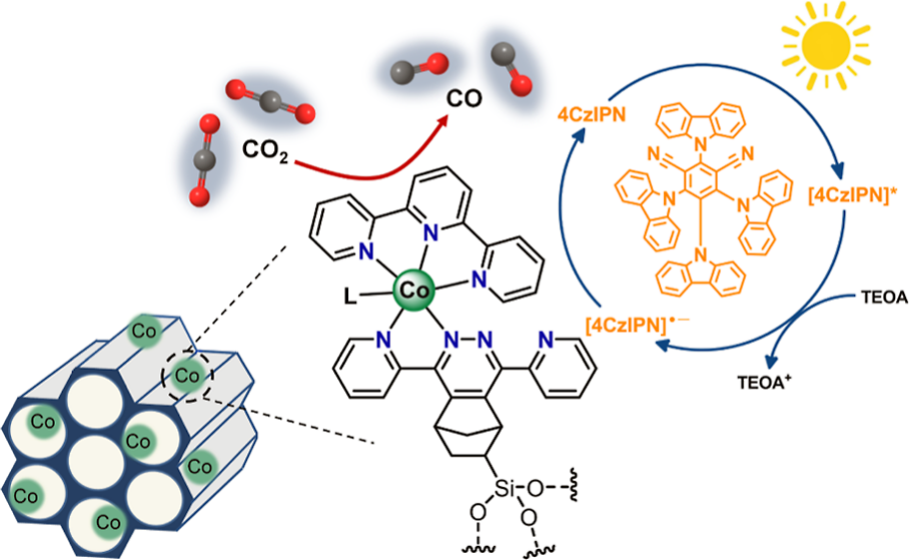

Photocatalytic CO_2_ reduction, including reaction rate, product selectivity, and longevity, is highly sensitive to the coordination structure of the catalytic active sites, and the precise design of the active site remains a challenge in heterogeneous catalysts. Herein, we report on the modulation of the coordination structure of MN_*x*_-type active sites (M = Co or Ni; *x* = 4 or 5) anchored on a periodic mesoporous organosilica (PMO) support to improve photocatalytic CO_2_ reduction. The PMO was functionalized with pendant 3,6-di(2′-pyridyl)pyridazine (dppz) groups to allow immobilization of molecular Co and Ni complexes with polypyridine ligands. A comparative analysis of CO_2_ photoreduction in the presence of an organic photosensitizer (4CzIPN, 1,2,3,5-tetrakis(carbazol-9-yl)-4,6-dicyanobenzene) and a conventional [Ru(bpy)_3_]Cl_2_ sensitizer revealed strong influence of the coordination environment on the catalytic performance. CoN_5_-PMO demonstrated a superior CO_2_ photoreduction activity than the other materials and displayed a cobalt-based turnover number (TON_CO_) of 92 for CO evolution at ∼75% selectivity after 3 h irradiation in the presence of 4CzIPN. The hybrid CoN_5_-PMO catalyst exhibited better activity than its homogeneous [CoN_5_] counterpart, indicating that the heterogenization promotes the formation of isolated active sites with improved longevity and faster catalytic rate.

## Introduction

The photocatalytic valorization of CO_2_, in particular through its transformation into CO or syngas, represents an appealing CO_2_ mitigation strategy that has the potential to significantly contribute toward net-zero carbon economy. The use of homogeneous or semiheterogeneous colloidal photosystems, combining a photosensitizer with a CO_2_ reduction cocatalyst, has emerged as a simple and effective approach in solar-driven CO_2_ conversion technology. However, these hybrid photosystems often have several shortcomings such as expensive noble-metal-based sensitizers and cocatalysts, low photostability of sensitizers and/or cocatalysts, poor recyclability, and limited visible light absorption, restricting their implementation in practical systems. From a sustainability perspective, it is important to design photosystems that use organic dyes or non-noble metal-based sensitizers for harvesting the solar energy. However, in comparison to the Ru- and Ir-based molecular sensitizers, which are ubiquitous in photocatalytic CO_2_ reduction literature, organic dyes are relatively underutilized as photosensitizers. A few recent reports on precious-metal-free photosystems have demonstrated the viability of organic dyes, such as purpurin,^1,2^ phenoxazine,^3^ anthraquinone derivatives,^4^ and thermally activated delayed fluorescence (TADF) compounds,^5−7^ but generally, the scope of the catalysts that can be combined with the organic dyes is quite narrow. Therefore, it is important to expand the library of precious-metal-free photosystems by developing new catalyst/dye combinations for CO_2_ photoreduction.

From a catalyst design perspective, a wide range of molecular catalysts based on transition metal complexes have been developed for application in CO_2_ photoreduction by coupling with molecular sensitizers or light harvesting semiconductors.^8−11^ While molecular catalysts offer distinct benefits such as better atom efficiency, high product selectivity, tunability, and scope for mechanistic understanding, they often suffer from poor stability and are nonrecyclable when used under homogeneous conditions. However, these shortcomings can be mitigated *via* heterogenization of the molecular complexes on solid supports,^12−15^ which bridges between homogeneous and heterogeneous systems and provides an opportunity to precisely regulate the structure of the active site on solid support at atomic levels. The primary coordination environment around the central metal-active site plays a crucial role in controlling the catalytic reaction. MN_*x*_-type catalytic sites with a transition metal center coordinated with multiple N atoms are one of the most classical active sites in CO_2_ photoreduction, and the catalytic activity of these systems is highly dependent on the nature of the metal site, number of N donors, type of the coordinating N atoms, and the electronics of the ligands.^16−19^ MN_*x*_-type active sites are frequently reported in single-atom catalysts on various support materials including carbon nitride, graphene, and metal oxides,^18−24^ but fine-tuning the coordination structure of the integrated MN_*x*_ sites is often challenging. In this context, we report a strategy for controlling the molecular structure of metal-active sites grafted on functionalized periodic mesoporous organosilica (PMO) support to optimize CO_2_ photoreduction. Notably, the porous support plays an important role by allowing diffusion of the reactants and reagents to access the molecular active sites and by regulating the local chemical environment during catalysis.^25^

PMOs, synthesized from organo-bridged alkoxysilane precursors by a directing surfactant self-assembly approach, are one of the most representative organic–inorganic hybrid materials with the organic functionalities homogeneously distributed within the pores and the walls of the silica framework.^26^ Due to their unique characteristics such as ordered mesostructures with adjustable pore size, high surface areas, and tunable hydrophobicity/hydrophilicity, PMOs have been demonstrated to be promising scaffolds to construct heterogeneous photocatalytic systems for CO_2_ reduction.^27,28^ Most of the studies reported in this field have been focused on the use of the Bpy-PMO (bipyridine-functionalized PMO) platform to immobilize metal complexes.^29−31^ The surface bipyridine groups serve as chelating ligands for the formation of metal complexes on the surface pores, thus resulting in the formation of isolated active sites. Through this synthetic approach, heterogeneous molecular Re, Ru, and Mn catalysts have been successfully constructed on the surface of Bpy-PMO for mediating CO_2_ photoreduction.^29−31^ Recently, catalytic performance was improved by using Bpy-PMO with a tubular structure that facilitated the diffusion of the reactants through the large pores, leading to faster photocatalysis and enhanced quantum yields.^32^ As an alternative to the Bpy-PMO platform to immobilize metal complexes, recently our group reported the synthesis of a novel dipyridyl-pyridazine triethoxysilane precursor (Ndppz) through a facile and rapid approach based on an inverse electron demand Diels–Alder (iEDDA) reaction.^33^ By co-condensation reactions of the Ndppz precursor with 1,2-bis(triethoxysilyl)ethane (BTEE), we achieved a novel PMO material with surface-attached pendant N-chelating heterocyclic ligands, 3,6-di(2′-pyridyl)pyridazine (dppz). Ndppz-PMO was successfully utilized as a solid chelating ligand to immobilize Ru and Ir complexes and generate light-harvesting materials for the photocatalytic hydrogen evolution reaction. Very recently, we have also reported the synthesis of a dppz-functionalized mesoporous SBA material and its use as a heterogeneous water oxidation catalyst after complexation with IrCp*Cl.^34^ These results demonstrate the great potential of heterogenized N-chelating heterocyclic ligands as a novel platform for the construction of dppz-based solid MN_*x*_ catalysts.

Systemic analysis to rationally design the optimum coordination environment of MN_*x*_-type active sites toward CO_2_ photoreduction in heterogeneous colloidal suspensions has rarely been reported.^35^ Herein, the dppz-functionalized PMO (NdppzPMO) has been used as a versatile scaffold for installing MN_*x*_-type units on the surface and investigate the effect of changing the primary coordination sphere on photocatalytic CO_2_ reduction. Four single-site MN_*x*_-PMO catalysts (M = Co or Ni, *x* = 4, 5, *x* representing the number of pyridine N atoms coordinated with the metal center) were synthesized by anchoring [M(tpy)]^2+^ or [M(bpy)]^2+^ moieties (tpy = 2,2′:6′,2″-terpyridine and bpy = 2,2′-bipyridine). In CO_2_ photoreduction studies with a traditional Ru(bpy)_3_Cl_2_ photosensitizer (denoted as [Ru-PS]) and an organic TADF dye (4CzIPN), MN_*x*_-PMO catalysts displayed distinct CO evolution activity with CoN_5_-PMO exhibiting superior performance than the other three materials. 4CzIPN was selected as the sensitizer because it has been shown to be a cost-effective option for photoredox catalysis,^36^ and its use in CO_2_ reduction photosystems was recently demonstrated for molecular iron-terpyridine and manganese-bipyridine catalysts.^5−7,37−40^ The coordination environment around Co in CoN_5_-PMO was confirmed by X-ray absorption spectroscopy (XAS), and the origin of the activity at the CoN_5_-active site was supported by mechanistic studies on a molecular CoN_5_ analogue.

## Results and Discussion

### Synthesis and Characterization

**Figure 1:**
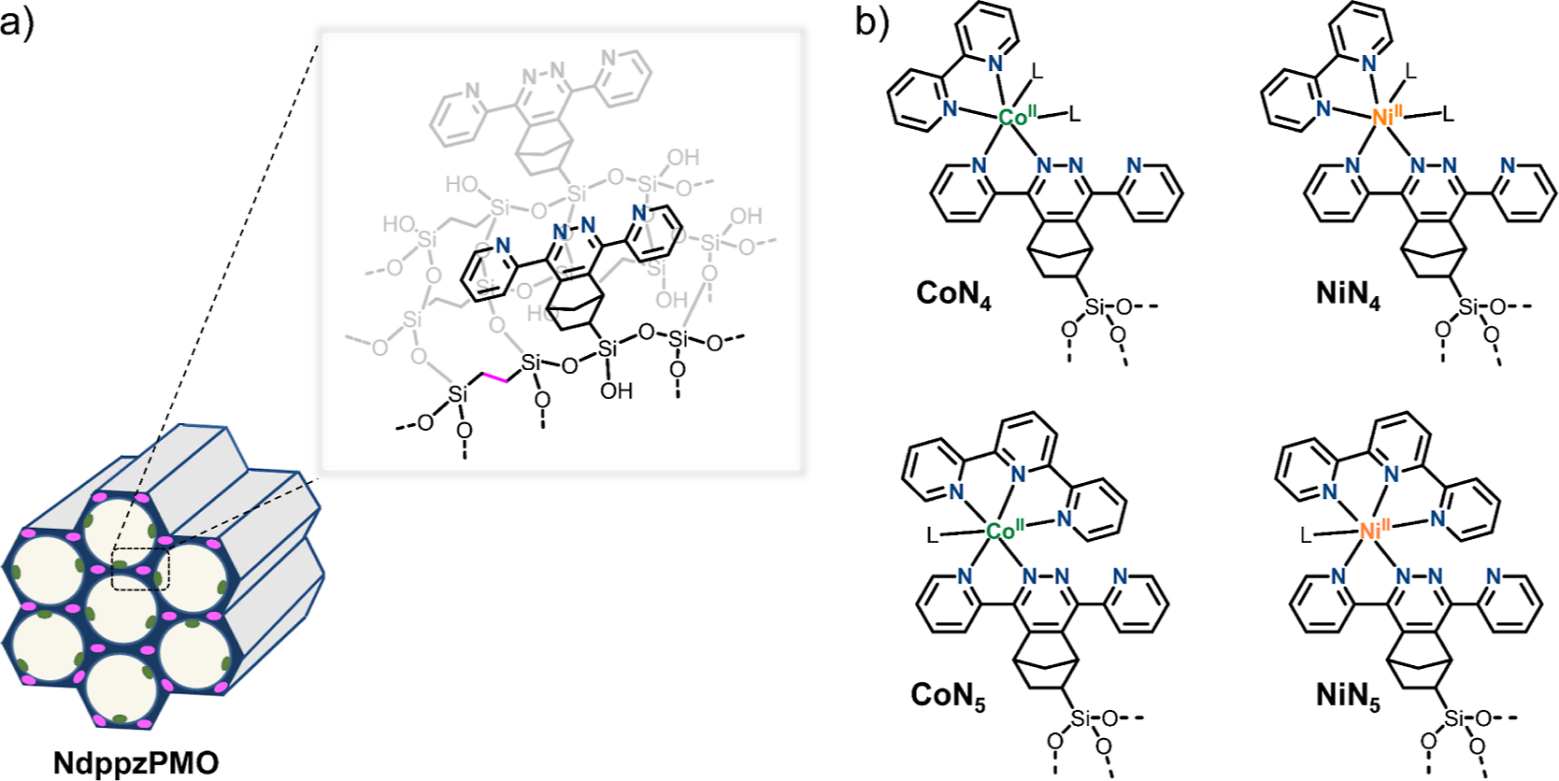
(a) Schematic structure of NdppzPMO. (b) Chemical structures of MN_*x*_ catalytic sites immobilized on NdppzPMO scaffold via surface-bound dipyridylpyridazine motifs.

**Figure 2:**
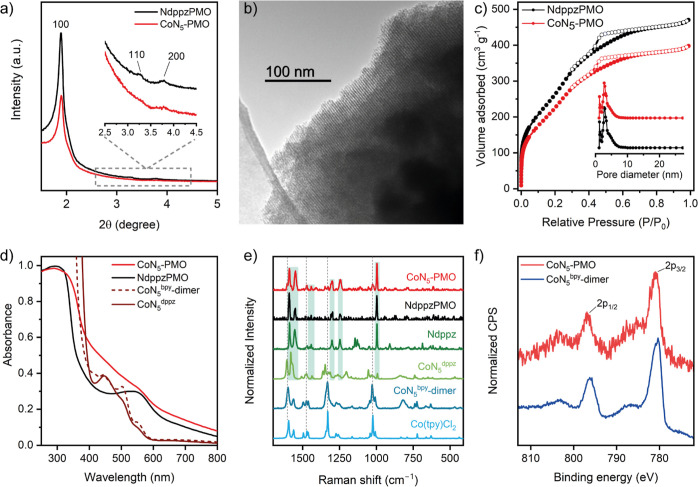
(a) Powder X-ray diffraction pattern of NdppzPMO and CoN_5_-PMO. (b) TEM image of CoN_5_-PMO. (c) N_2_ adsorption–desorption isotherms and PSD (inset) of NdppzPMO and CoN_5_-PMO. Adsorption and desorption branches are plotted with solid and open circles, respectively. (d) UV–vis diffuse reflectance spectra of NdppzPMO and CoN_5_-PMO and UV–vis absorption spectra of CoN_5_^dppz^ in MeCN (1.06 mM) (brown solid trace) and CoN_5_^bpy^ dimer in MeCN (0.39 mM) (brown dashed trace); (e) Raman spectra of CoN_5_-PMO, NdppzPMO, Ndppz, CoN_5_^dppz^, CoN_5_^bpy^ dimer, and Co(tpy)Cl_2_. Characteristic vibrational modes of Ndppz and Co(tpy) units are indicated with green shades and dashed lines, respectively, and (f) Co 2p XPS spectra of CoN_5_-PMO and CoN_5_^bpy^ dimer.

The PMO with pendant 3,6-di(2′-pyridyl)pyridazine (dppz) binding motifs was synthesized by co-condensation of the Ndppz trialkoxysilane-functionalized precursor (denoted as Ndppz, 30 mol %) and a conventional bis-silane precursor (BTEE, 70 mol %), in the presence of a cationic surfactant (Figure 1a).^33^ Successful synthesis of Ndppz-functionalized PMO (NdppzPMO) was confirmed by powder X-ray diffraction and Raman spectroscopy (Figure 2). Molecular loading of 0.56 mmol dppz g^–1^ was determined from elemental analysis (3.15 wt % of N content). The assembly of different MN_*x*_-isolated single sites on NdppzPMO was accomplished by the coordination of the [M(tpy)] and [M(bpy)] units (tpy = 2,2’:6′,2″-terpyridine and bpy = 2,2′-bipyridine) onto the surface N-chelating motifs (Figure 1b). The hybrid PMOs are defined as MN_*x*_-PMO. The postsynthetic metalation of NdppzPMO with the molecular precursors (M(tpy)Cl_2_ and M(bpy)Cl_2_) promoted a change of color of the solid from pale pink to light brown, suggesting successful formation of MN_*x*_-PMO. The metal loading in the materials was determined by inductively coupled plasma-optical emission spectroscopy (ICP-OES) (Table S1). As a control material, CoN_2_-PMO was synthesized by metalation of NdppzPMO with CoCl_2_, producing cobalt centers coordinated to the dppz units in PMO.

Preliminary screening of the four MN_*x*_-PMOs as photocatalysts for CO_2_ reduction in the presence of [Ru-PS] or 4CzIPN as a photosensitizer and triethanolamine (TEOA) as a sacrificial electron donor demonstrated superior performance of CoN_5_-PMO (vide infra). Subsequently, the in-depth characterization of the hybrid material was focused on CoN_5_-PMO. ICP-OES analysis of CoN_5_-PMO showed a cobalt loading of 0.041 μmol Co mg^–1^, which was consistent with the 0.035 μmol tpy mg^–1^ estimated by ^1^H NMR of acid-digested CoN_5_-PMO (Figure S1). This data indicates that only ∼7% of the available dppz ligands were coordinated with Co(tpy) units, suggesting that the remaining uncoordinated dppz ligands have lower accessibility.

The preservation of the periodic mesoporous structure after the formation of surface CoN_5_ sites was confirmed by low-angle X-ray diffraction, transmission electron microscopy, and nitrogen adsorption–desorption isotherms. Powder X-ray diffraction pattern of CoN_5_-PMO showed characteristic lattice planes of a *P*6 *mm* hexagonal arrangement structure with a main reflection at 2θ value of 1.90° (an interplanar spacing (*d*_100_) of 4.6 nm) analogous to the pristine material (Figure 2a).^41^ However, differences in the scattering contrasts within the pores were observed due to the decrease in the intensity of the diffractogram obtained after Co(tpy) coordination.^34^ XRD results were supported by TEM images, which showed a highly ordered structure with the pore channel axis oriented parallel to the electron beam (Figure 2b). The porosity of CoN_5_-PMO was evaluated by nitrogen physisorption measurements, obtaining a nitrogen adsorption–desorption isotherm with capillary condensation in the *P*/*P*_0_ range from 0.3 to 0.7 (Figure 2c). The isotherm exhibited largely type IV behavior and a hysteresis loop of type H2, typical of ordered mesoporous materials. In addition, the pore size distribution (PSD) also indicated the presence of microporosity (Figure 2c—inset).^42^ Successful immobilization of the Co complex within PMO cavities was confirmed by a decrease in Brunauer–Emmett–Teller surface area (*S*_BET_) from 901 to 750 m^2^ g^–1^ and pore volume (*V*_P_) from 0.72 to 0.60 cm^3^ g^–1^. However, the pore diameter (*D*_P_) remained unchanged, with an average value of 3.2 nm.

Surface functionalization of NdppzPMO with CoN_5_ sites was corroborated by UV–vis diffuse reflectance spectroscopy (UV–vis DRS) and Raman spectroscopy, while local electronic and atomic structures around the cobalt center were assessed by XAS and X-ray photoelectron spectroscopy (XPS). As shown in Figure 2d, the UV–vis DRS of CoN_5_-PMO exhibited a characteristic π–π* band at 290 nm originating from the dppz and the terpyridine ligands^43,44^ and an n–π* band at 540 nm for the pyridazine units. In addition, coordination of the metal complex led to the appearance of a broad shoulder at around 450 nm ascribed to metal-to-ligand charge transfer (MLCT). This finding was corroborated with similar absorptions observed in the UV–vis spectra of the homogeneous analogue (Figure 2d) and Co(tpy)-based complexes.^45^ Immobilization of Co(tpy) in CoN_5_-PMO was further confirmed by Raman spectroscopy, which showed characteristic Si–O stretching vibration at 995 cm^–1^, intense C=N vibrations at 1590 cm^–1^, and additional signals at 1549, 1300, and 1245 cm^–1^ originating from various skeletal vibrations of pyridine/pyridazine heterocycles (Figure 2e).^33^ New signals were observed at 1601, 1471, 1330, and 1026 cm^–1^ that can be attributed to the anchored Co(tpy) moiety.^46,47^ Furthermore, the absence of symmetric ν_O–O_ stretching vibration for μ-O_2_ species at 821 cm^–1^ confirms the formation of monomeric CoN_5_ species. Thermogravimetric analysis (TGA) of CoN_5_-PMO showed two clear weight loss steps: (1) ∼ 8% loss below 100 °C from evaporation of solvent(s) and (2) ∼ 7% loss at 390–450 °C likely due to thermal degradation of the organic content (Figure S2). The weight loss profile of CoN_5_-PMO was similar to that for NdppzPMO, consistent with the unchanged underlying structure of the PMO support.

**Figure 3:**
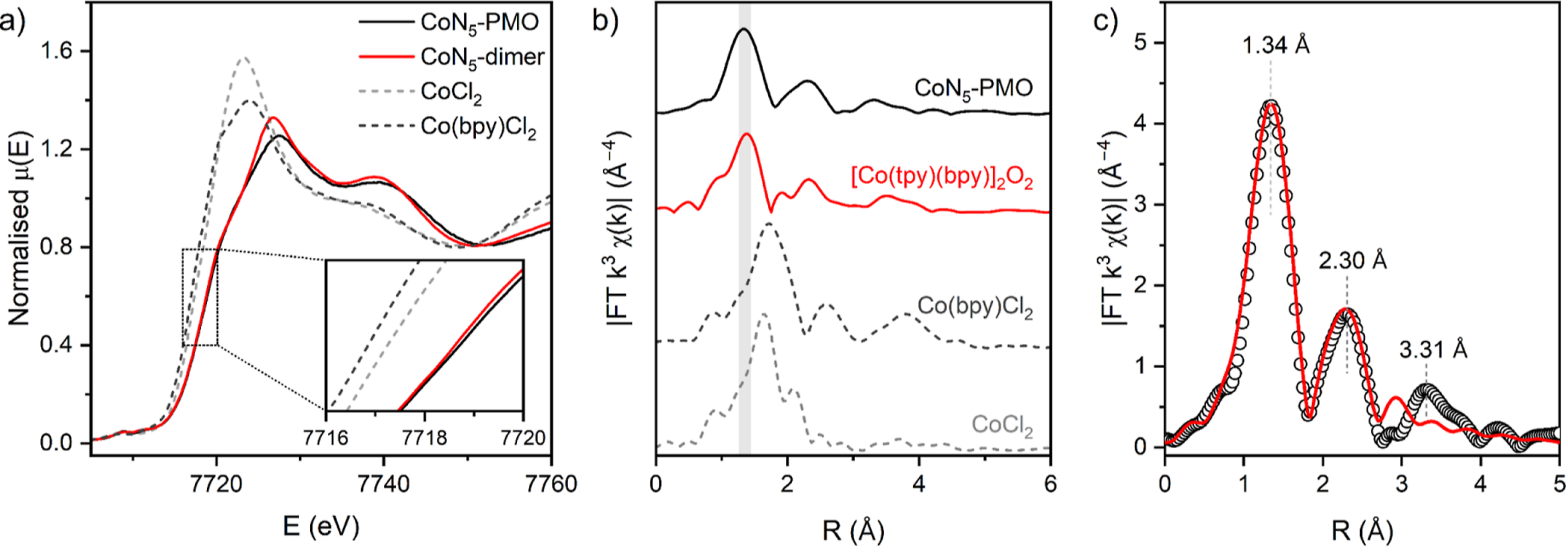
X-ray absorption data: (a) Co K-edge XANES spectra and (b) Fourier transform R-space EXAFS data of CoN_5_-PMO, CoN_5_^bpy^ dimer, Co(bpy)Cl_2_, and CoCl_2_; and (c) EXAFS fitting of CoN_5_-PMO with the data shown as open circles and the FEFF fit as a solid red line.

XPS measurements were performed to probe the surface composition of PMO and the electronic structure of the CoN_5_ sites (Figures 2f and S3). The survey spectrum of CoN_5_-PMO showed the presence of Si, O, C, N, and Co (Figure S3a). In the Co 2p region, two peaks were observed at 780.9 eV (Co 2p_3/2_) and 796.8 eV (Co 2p_1/2_) (Figure 2f), which was in good agreement to the peaks observed for the reference molecular dimeric Co(III) complex, [{Co(tpy)(bpy)}_2_(μ-O_2_)](PF_6_)_4_ (abbreviated as CoN_5_^bpy^ dimer), demonstrating the +3 oxidation state of Co. The weak shakeup satellite peaks for Co are also consistent with a predominantly diamagnetic Co^3+^ species. The N 1s region of the XPS for CoN_5_^bpy^ dimer features a peak at 399.6 eV, which is similar to that observed for CoN_5_-PMO and previously reported systems based on NdppzPMO.^33^ Deconvolution of the N 1s peak showed two signals at 399.9 and 399.1 eV (Figure S3d), which can be attributed to Co–N bonds and noncoordinated pyridine/pyridazine N, respectively. To further resolve the local coordination environment around the cobalt center, steady-state XAS was performed at the Diamond Light Source (Figures 3, S4 and S5). The X-ray near-edge spectroscopy (XANES) spectra for CoN_5_-PMO at Co K-edge showed a very similar position and shape of the absorption edge to that for the CoN_5_^bpy^ dimer,^48^ indicating that the cobalt centers in PMO are in an octahedral geometry with five pyridinic ligands and a coordinated solvent molecule. The first derivative of XANES spectra showed excellent agreement between the edge energy (*E*_0_) of CoN_5_-PMO and CoN_5_^bpy^ dimer, while the two Co(II) reference compounds displayed edge position at lower energy, which is consistent with a +3 oxidation state of the Co centers in PMO (Figure S4b). Fourier transform-extended X-ray absorption fine structure (FT-EXAFS) spectrum of CoN_5_-PMO in the R-space showed a dominant peak at ∼1.34 Å and two weaker peaks at 2.30 and 3.31 Å (Figure 3b). The first peak at a shorter radial distance originates from the single-scattering Co–N/O pathways in the first coordination shell. The scattering paths visible at higher *R* values (*R* = 2.30 and 3.31 Å) originate from the second and third coordination shell of tpy and dppz ligands and include Co–C/N single-scattering and Co–N–C/Co–C–C multiple-scattering paths (Figure 3b,3c). The close similarity between the R-space EXAFS data for CoN_5_-PMO and CoN_5_^bpy^ dimer suggests an analogous coordination environment around the Co center in PMO with five N donors from tpy and dppz ligands and one O/N donor from a solvent molecule (H_2_O or MeCN). In comparison, the major peak for the first coordination shell of the two Co(II) reference compounds (Co(bpy)Cl_2_ and CoCl_2_) appeared at longer radial distances, consistent with the +2 oxidation state of Co. To gain further details of the molecular structure of the Co site, EXAFS fittings were performed using the FEFF input model built from the crystal structure of the CoN_5_^bpy^ dimer, by changing the bipyridine ligand to dppz. The best fit in the R-space and *k*-space for the first and second coordination shells is shown in Figures 3c and S5, respectively. The fitting parameters are listed in Table S2, and the structural model is shown in Figure S6. The best fit indicates that the first coordination shell consists of four Co–N bonds with a 1.97 Å bond distance, another Co–N bond at 1.80 Å, and a coordinated water molecule (or a hydroxide ligand) with a 1.82 Å long Co–O bond. The shorter Co–N distance corresponds to the bond between Co and the central nitrogen atom of the tpy ligand. The Co–N/O bond distances are consistent with a Co(III) center and are within the range of distances reported in the literature,^48,49^ validating our fitting model. The second EXAFS peak at 2.30 Å was fitted with a second shell of single-scattering paths originating from the tpy and dppz ligands, which consists of six Co–C single-scattering paths at 2.86 Å, a Co–N path at 2.99 Å, and another Co–C path at 3.08 Å. Importantly, the fit suggests the absence of Co–Co scattering paths, confirming the molecular nature of single Co(III) sites in the hybrid PMO material (Figure 3c). Using similar fitting procedures, the coordination structure of the Co sites in CoN_4_-PMO was also evaluated based on the EXAFS fit with a structural model of Co(bpy)(dppz)(OH_2_)_2_ (Figures S4 and S5), and the results are summarized in Table S3.

To corroborate the CO_2_ reduction capability of the CoN_5_-active sites on PMO, the molecular analogue, CoN_5_^bpy^ dimer, was probed in solution by using cyclic voltammetry (Figure S7). All potentials are reported against the ferrocene couple (Fc^+/0^). In N_2_-purged acetonitrile solution, the dimeric Co complex dissociates to form mononuclear [Co(tpy)(bpy)]^2+^ species (denoted as [CoN_5_^bpy^]^2+^), presenting close structural model of the active site in CoN_5_-PMO.^48^ Cyclic voltammogram of [CoN_5_^bpy^]^2+^ under N_2_ showed two reversible reductions at *E*_1/2_ = −0.12 and −1.17 V, attributable to Co^III/II^ and Co^II/I^ processes.^50^ At further reducing potentials, two overlapping reductions were observed with an irreversible process at −1.95 V (peak potential) and a quasi-reversible process at −2.05 V (*E*_1/2_), which can be assigned to ligand-centered reductions and/or Co^I/0^ reduction. Under CO_2_ saturation, current enhancement was observed near the third reduction process (onset potential −1.83 V and midwave potential −1.95 V), indicative of electrocatalytic CO_2_ reduction. In the presence of 10% (v/v) triethanolamine (TEOA) as a proton source, the onset of catalysis undergoes an anodic shift to −1.70 V, indicating that TEOA facilitates CO_2_ reduction at the Co-active sites. To further investigate the impact of replacing bpy with dppz ligand on CO_2_ reduction, a second molecular cobalt complex was synthesized bearing tpy and dppz ligands, [Co(tpy)(dppz)Cl](PF_6_)_2_ (denoted as CoN_5_^dppz^). Cyclic voltammograms of CoN_5_^dppz^ recorded in the CO_2_-saturated MeCN/TEOA (10% v/v) electrolyte showed an earlier onset of catalysis at −1.51 V, while the reduction potential for the two Co-centered process remained unchanged at −0.11 V (Co^III/II^) and −1.17 V (Co^II/I^) (Figures S8 and S9). This suggests that dppz ligand-centered reduction processes are involved in the catalytic reduction of CO_2_, and more electron-deficient nature of dppz enables an earlier onset of catalysis compared to the CoN_5_^bpy^ analogue.

### Photocatalytic CO_2_ Reduction

Since polypyridyl complexes of Co and Ni are well-known CO_2_ reduction catalysts,^51−56^ the four MN_*x*_-PMOs with distinct isolated single sites (CoN_4_-PMO, CoN_5_-PMO, NiN_4_-PMO, and NiN_5_-PMO) were initially screened for photocatalytic CO_2_ reduction in the presence of [Ru-PS] and TEOA donor. In a typical experiment 1 mg of MN_*x*_-PMO was dispersed in a CH_3_CN/TEOA (9:1) mixture containing the photosensitizer, purged with CO_2_ to saturate the colloidal suspension, and irradiated under UV-filtered simulated solar light (λ > 400 nm, 100 mW cm^–2^, AM 1.5G). Gas chromatography of the headspace showed that all the four materials produced CO and H_2_ (Table 1, entries 1–4). No liquid product was observed by ^1^H NMR spectroscopy or ion chromatography. Among the four catalysts, CoN_5_-PMO exhibited the best activity with a CO evolution of 2.13 μmol mg^–1^, a CO selectivity of 64.2%, and a corresponding turnover number (TON_CO_) of 52 after 1 h based on cobalt loading. Enhanced CO evolution and better CO selectivity were observed for CoN_*x*_-PMOs when BIH was introduced in the system as a two-electron donor, consistent with previous reports (Table 1, entries 5–8).^57,58^ In contrast, NiN_*x*_-PMOs displayed lower CO evolution in the presence of BIH. It should be noted that in the presence of BIH donor, TEOA mainly functions as a proton acceptor from BIH^+•^. TEOA is also known to form a zwitterionic alkylcarbonate adduct with CO_2_, which is a good proton donor.^59^ The preliminary results with PMO materials showed promising activity toward CO_2_ photoreduction, but the use of a noble-metal-based photosensitizer was a drawback, and metal-free organic photosensitizers were explored as potential alternatives for photocatalysis.

**Table 1:** Photocatalytic CO_2_ Reduction of MN_*x*_-PMO Catalysts in the Presence of [Ru-PS] or 4CzIPN (PS = Photosensitizer)^a^

entry	PS	catalyst	e^–^ donor	product (μmol mg^–^^1^)		CO selectivity^c^ (%)	TON_CO_^d^
				CO	H_2_		
1	[Ru-PS]	CoN_4_-PMO	TEOA	0.76 ± 0.01	1.19 ± 0.02	40	11
2		CoN_5_-PMO		2.13 ± 0.08	1.19 ± 0.08	64	52
3		NiN_4_-PMO		0.42 ± 0.11	0.59 ± 0.03	41	2.3
4		NiN_5_-PMO		0.95 ± 0.04	0.56 ± 0.02	63	5.9
5	[Ru-PS]	CoN_4_-PMO	BIH^b^	1.46 ± 0.11	0.84 ± 0.08	64	21
6		CoN_5_-PMO		3.70 ± 0.39	0.51 ± 0.01	88	90
7		NiN_4_-PMO		0.32 ± 0.07	0.60 ± 0.08	35	1.8
8		NiN_5_-PMO		0.47 ± 0.08	0.11 ± 0.01	81	2.9
9	4CzIPN	CoN_2_-PMO	TEOA	0.86 ± 0.11	0.55 ± 0.01	61	3
10		CoN_4_-PMO		0.97 ± 0.02	0.57 ± 0.03	63	14
11		CoN_5_-PMO		2.54 ± 0.17	1.03 ± 0.06	71	62
12		NiN_4_-PMO		0.01 ± 0.01	0.64 ± 0.04	1	
13		NiN_5_-PMO		0.80 ± 0.01	0.12 ± 0.02	41	5
14	4CzIPN	CoN_4_-PMO	BIH^b^	0.32 ± 0.09	0.33 ± 0.01	48	5
15		CoN_5_-PMO		0.50 ± 0.03	0.15 ± 0.00	77	12
16		NiN_4_-PMO			0.27 ± 0.03	0	
17		NiN_5_-PMO		0.07 ± 0.00		100	0.4
18	4CzIPN	CoN_5_dppz^e^	TEOA	0.83 ± 0.01^e^	0.30 ± 0.05^e^	73	20.9

a Reaction conditions: 1 mg of MN_*x*_-PMO, 4 mL of MeCN/TEOA (9:1 v/v), 0.5 mM photosensitizer ([Ru-PS] or 4CZIPN), visible light (100 mW cm^2^, AM 1.5G, λ > 400 nm), and 1 h irradiation.

b 10 mM BIH in 9:1 MeCN/TEOA.

c CO selectivity (%) = .

d TON_CO_ (after 1 h) =  (M = Co or Ni, *x* = 4 or 5).

e 0.041 μmol of CoN_5_^dppz^ in 4 mL of MeCN/TEOA (9:1 v/v) containing 0.5 mM 4CzIPN was used for photocatalysis, and the total amount of CO and H_2_ evolved after 1 h irradiation is reported in the table.

Three different organic dyes (9-cyanoanthracene, purpurin or 4CzIPN) were subsequently screened for photocatalytic activity, but CO evolution was observed only in the presence of 4CzIPN. Under this condition, CoN_5_-PMO outperformed the other catalysts with a CO evolution of 2.54 ± 0.17 μmol mg^–1^, a CO selectivity of 71%, and a TON_CO_ of 62 after 1 h irradiation (Table 1, entries 9–13). CoN_2_ and CoN_4_-PMO produced less CO at lower selectivity compared to CoN_5_-PMO. This result confirms that the Co centers coordinated with five N atoms provide the optimum active site structure for effective CO_2_ photoreduction. In comparison, when the mononuclear molecular cobalt complex (CoN_5_^dppz^) was employed as a homogeneous catalyst under identical conditions, a considerably slower CO evolution was observed with a TON_CO_ of 20.9 after 1 h, highlighting the benefits of heterogenizing CoN_5_ sites on PMO support toward facilitating CO_2_ photoreduction (Table 1, entry 18). Notably, the catalytic activity dropped to only 0.50 ± 0.03 μmol of CO mg^–1^ when BIH was used as the donor with 4CzIPN (Table 1, entry 15), indicating that BIH is not effective at quenching photoexcited 4CzIPN. Control experiments demonstrate that all components including CoN_5_-PMO, 4CzIPN, TEOA, visible light, and CO_2_ are required for photocatalysis, and a negligible amount of CO was evolved in the absence of any single component (Table S4). Interestingly, the CoN_5_-PMO/4CzIPN/TEOA photosystem produced 6.23 μmol H_2_ mg^–1^ under N_2_, corresponding to a turnover number (TON_H2_) of ∼152. This indicates that the CoN_5_-active site is capable of catalyzing H_2_ evolution, but the product selectivity is tuned toward CO under CO_2_-saturated conditions.

**Figure 4:**
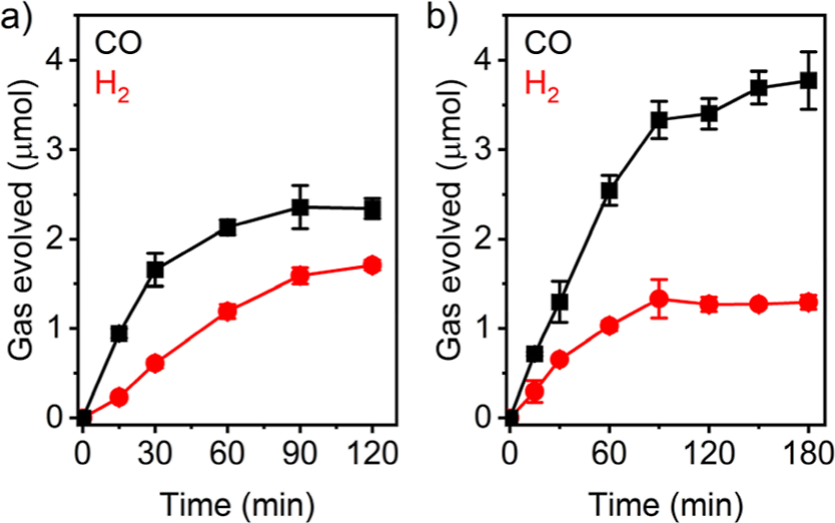
Time dependence of CO evolution (black trace) and H_2_ evolution (red trace) for CoN_5_-PMO in CO_2_-saturated MeCN/TEOA (9:1 v/v) in the presence of (a) [Ru-PS] and (b) 4CzIPN.

**Figure 5:**
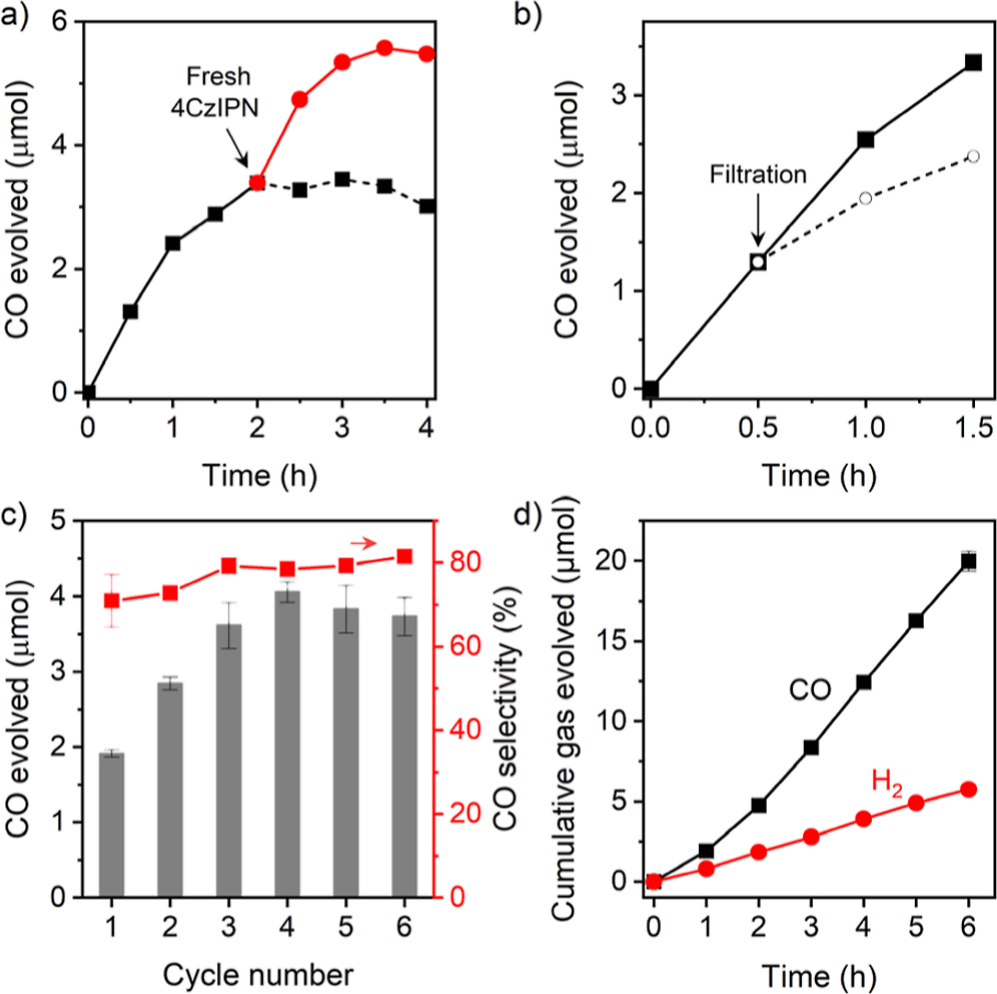
(a) Reactivation of photocatalysis mixture by addition of fresh 0.5 mM 4CzIPN (red trace). Black dashed line demonstrates plateauing of the activity after 2 h of irradiation. (b) Filtration experiment for CoN_5_-PMO to distinguish the activity of the solid catalyst and active species in the solution. Solid line shows the normal course of the reaction using colloidal CoN_5_-PMO. The dashed line shows the activity of the filtrate collected after 30 min irradiation. (c) Recycling experiment for CoN_5_-PMO (5 mg) showing CO evolution and CO selectivity during six 1 h photocatalysis experiments. (d) Cumulative amount of CO and H_2_ evolved over the course of 6 h recycling experiments.

Photocatalysis experiments with CoN_5_-PMO over longer duration showed that the CO evolution started to plateau after 2 h for both [Ru-PS]/TEOA and 4CzIPN/TEOA combinations (Figure 4). The amounts of CO produced at the plateau were 2.35 ± 0.11 and 3.78 ± 0.32 μmol mg^–1^ for [Ru-PS] and 4CzIPN, respectively, corresponding to Co-based TON_CO_ of 57 and 92. For [Ru-PS], the CO selectivity peaked at 81% after 15 min, followed by gradual decrease over 2 h due to the enhanced H_2_ evolution catalyzed by [Ru-PS] photodegradation products.^60,61^ In contrast, the CO selectivity remained largely constant at 70–75% when 4CzIPN was employed, suggesting that CoN_5_-PMO retained the CO_2_ reduction activity, and the longevity of the photocatalytic process was limited by the stability of photosensitizers. This hypothesis was confirmed by the addition of fresh 4CzIPN into the reaction mixture after CO evolution had ceased, which reactivated the system and resumed the CO evolution (Figure 5a). Notably, marked improvement in performance was observed when BIH was added as a donor to the [Ru-PS]/TEOA system, leading to evolution of 5.76 ± 0.47 μmol of CO mg^–1^ at 87% CO selectivity after 2 h (Figure S10). However, significant CO evolution was observed after 2 h in the control experiment with a [Ru-PS]/TEOA/BIH mixture without CoN_5_-PMO, caused by the Ru-based decomposition products. Therefore, we focused on the metal-free 4CzIPN/TEOA combination for subsequent studies due to its robust activity and noncatalytic photodegradation products.

The photocatalytic activity was optimized by varying the catalyst/photosensitizer ratio. At fixed concentrations of TEOA (10% v/v) and CoN_5_-PMO (1 mg), increasing the 4CzIPN concentration enhanced the overall rate of CO production (Figure S11a). Early deactivation of the system was observed at lower concentration of 4CzIPN, as demonstrated by bleaching of the yellow color and plateauing of CO evolution. The amount of CO evolved during the initial 15 min irradiation showed a linear correlation with the concentration of 4CzIPN, suggesting a pseudo first-order dependence (Figure S11b). When the 4CzIPN concentration was fixed at 0.5 mM and the amount of CoN_5_-PMO was varied from 1 to 3 mg, a similar rate of CO evolution was observed with the highest TON_CO_ obtained at low catalyst loading (Figure S12). This suggests that the activity of the system is limited by electron transfer to the catalyst. Moreover, light scattering in concentrated colloidal suspension can also impede the photocatalytic activity.^62^ The apparent quantum yield (AQY) for CO evolution by the CoN_5_-PMO/4CzIPN/TEOA photosystem was determined to be 0.51% at monochromatic irradiation of 467 nm, using ferrioxalate as a chemical actinometer (Figures S13 and S14).^63^

The heterogeneous nature of the photocatalytic system was investigated by six 1 h recycling experiments using a higher loading of CoN_5_-PMO (5 mg) to minimize the impact of material loss during workups between cycles. The CO evolution gradually increased with the consecutive recycling runs, with the activity reaching a maximum during the fourth cycle, followed by a slow decrease in the subsequent runs. The CO selectivity increased from first to third cycle and remained largely unchanged afterward at ∼80% (Figure 5c). A similar slow activation behavior was also reported in photocatalytic CO_2_ reduction by cobalt phthalocyanine-modified mesoporous carbon nitride.^64^ For CoN_5_-PMO, the cumulative amount of CO produced over six recycling runs showed slightly slower rate during 0–2 h and a near-linear CO evolution rate during the subsequent 4 h photocatalysis (Figure 5d). Linear fit of the gas evolved during second to sixth runs yields a CO evolution rate of 3.82 μmol h^–1^ and a turnover frequency (TOF_CO_) of 19.3 h^–1^ for 5 mg of CoN_5_-PMO (Figure S15). It should be noted that TOF_CO_ is determined based on the initial Co loading and does not account for any loss of Co due to leaching. This result confirms the heterogeneous nature of the catalysis and suggests that the longevity of the system is likely limited by the photodegradation of 4CzIPN. Since the photosensitizer was replenished after each recycling run, sustained CO_2_ photoreduction was observed with CoN_5_-PMO. Interestingly, ICP-OES analysis of CoN_5_-PMO after the recycling experiment showed a Co loading of 0.018 μmol mg^–1^, indicating a loss of 56% Co during the six recycling runs. To corroborate this data, a leaching test was performed by filtering the reaction mixture after 30 min of irradiation and retesting the filtrate for photocatalytic activity. Irradiation of the CO_2_-saturated filtrate showed lower CO evolution rate, suggesting that contributions from both homogeneous and heterogeneous catalysts were present in the photosystem (Figure 5b). The homogeneous contribution likely came from the [Co(tpy)] fragments in solution that decoordinated from the dppz ligands on the PMO support. This result was supported by the photocatalytic activity displayed by Co(tpy)Cl_2_ under homogeneous conditions in the presence of 4CzIPN (1.28 μmol CO after 1 h, TON_CO_ = 31, 90% CO; Table S4, entry 11). Addition of unfunctionalized nondppz PMO to the homogeneous photosystem had minimal effect on CO evolution activity (Table S4, entry 12). In solution, Co(tpy)Cl_2_ can disproportionate to form [Co(tpy)_2_]^2+^,^65^ and therefore, the photolysate likely contained a mixture of both species that contribute toward CO evolution. However, the steady CO evolution rate observed during recycling experiments suggests that the contribution from heterogeneous catalysis is dominant. We hypothesize that the fraction of [CoN_5_]-active sites buried deeply within the mesopores can potentially become more accessible with longer exposure time, which can compensate for the Co loss from leaching and lead to a steady CO evolution rate. Another possibility is that all [CoN_5_] sites did not engage in catalysis due to the electron transfer step from photosensitizer to [CoN_5_] being rate limiting. Since a higher catalyst (5 mg)-to-sensitizer (0.5 mM) ratio was used in the recycling experiments, it is likely that only the more accessible [CoN_5_] sites engaged in catalysis during the initial runs. During the subsequent recycling runs, when some of these [CoN_5_] sites underwent deactivation due to loss of Co(tpy) fragments, the reaction rate was compensated by the pristine and intact [CoN_5_] units that did not engage in catalysis previously.

To evaluate structural changes during the photocatalytic reactions, the irradiated CoN_5_-PMO catalyst was characterized by low-angle XRD and TEM (Figure S16a,b), which demonstrated retention of the mesoporous structure after catalysis. The integrity of the catalytic centers was examined by Co 2p XPS surface analysis of the postirradiation catalyst, showing unaffected binding energies for Co 2p_3/2_ and Co 2p_1/2_ peaks at 780.9 and 796.8 eV, respectively, which suggested the preservation of the initial oxidation state for the remaining Co centers (Figure S16c). In addition, the partial loss of Co species during photocatalysis was confirmed with the decrease in the intensity of the Co 2p contributions in XPS and the reduction of both π–π* and MLCT transitions in comparison with the nonirradiated CoN_5_-PMO (Figure S16).

To gain mechanistic insights, the photosystem was probed by fluorescence and *in situ* UV–vis spectroscopy. The molecular complex, CoN_5_^bpy^ dimer, was used as a proxy for CoN_5_-PMO to perform these experiments under homogeneous conditions. Quenching experiments of a 4CzIPN solution excited at 400 nm demonstrated a clear decay of the emission at 560 nm in the presence of TEOA (Figure S17a). The reductive quenching rate constant (*k*_q_) was determined to be 9.6 × 10^9^ M^–1^ s^–1^ based on the Stern–Volmer equation, close to the diffusion limit (Figure S17b). In contrast, the oxidative quenching of photoexcited 4CzIPN* by CoN_5_^bpy^ dimer was minimal (Figure S18). Furthermore, the concentration of TEOA in the photocatalytic experiments was significantly higher than the catalyst concentration, and consequently, the reductive quenching of 4CzIPN* to form [4CzIPN]^•–^ is expected to be the first electron-transfer step. The reduced sensitizer, [4CzIPN]^•–^ (*E*_1/2_ (PS/PS^•–^) = −1.72 V), is a stronger reductant than the photoexcited sensitizer (*E*_1/2_ (PS*/PS^+^) = −1.04 V).^7,36^ Based on the electrochemical data for [CoN_5_^bpy^]^2+^ and [CoN_5_^dppz^]^2+^, [4CzIPN]^•–^ is sufficiently reducing to generate the reduced catalyst and prompt CO_2_ reduction in the presence of TEOA (onset potential −1.51 V).

**Figure 6:**
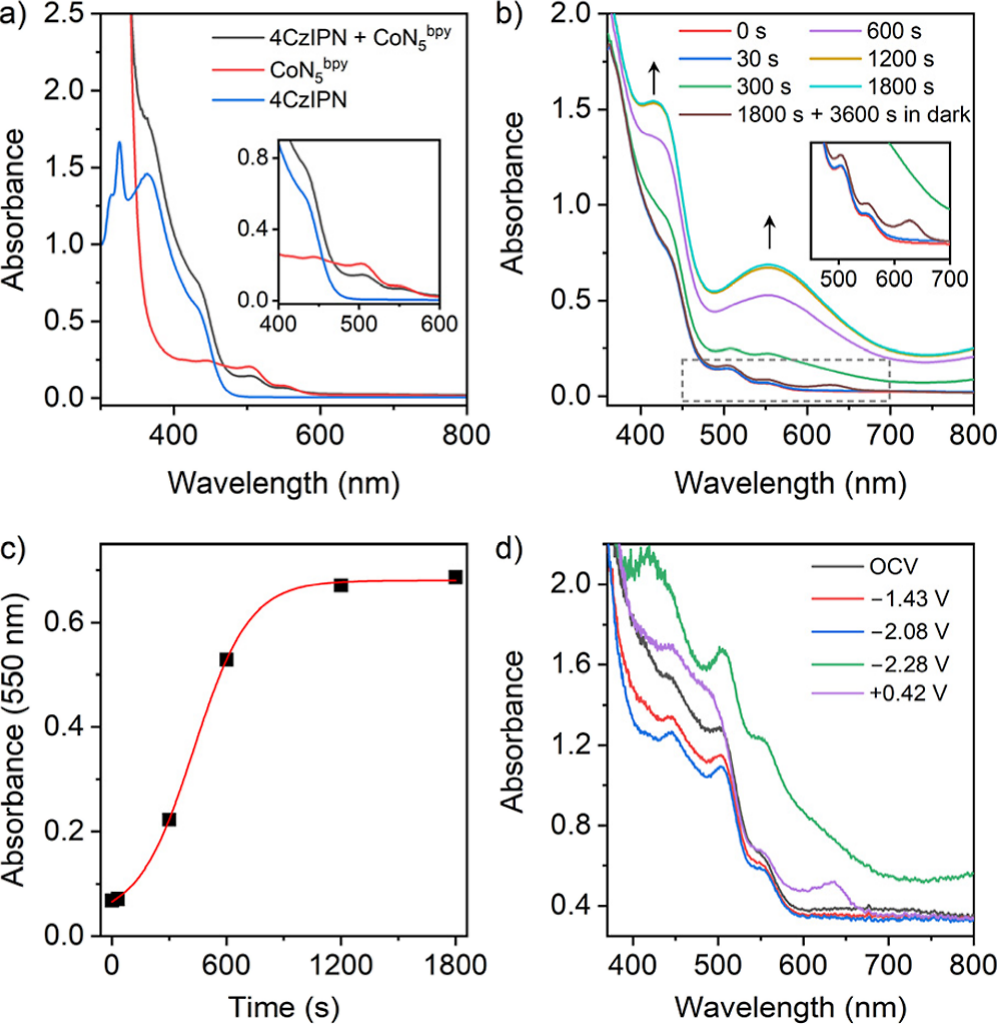
(a) UV–vis absorption spectra of 4CzIPN (0.1 mM), CoN_5_^bpy^ (0.11 mM), and their mixture in MeCN/TEOA (9:1 v/v). (b) Evolution of the UV–vis spectra of the photocatalysis mixture containing 4CzIPN (0.1 mM) and CoN_5_^bpy^ (0.11 mM) in CO_2_-saturated MeCN/TEOA (9:1 v/v) during visible light irradiation for 30 min, followed by 1 h storage in the dark. (c) Kinetic trace for the formation of photogenerated reduced species, demonstrated by the change in absorbance of the 550 nm band with irradiation time. The red line shows the sigmoidal fitted curve. (d) *In situ* UV–vis spectroelectrochemistry of the CoN_5_^bpy^ dimer (∼5 mM) in MeCN. The platinum working electrode was held at each potential for 2 min before recording the spectrum. The pre-electrolysis spectrum recorded at open-circuit voltage is shown in dark gray.

*In situ* UV–vis spectroscopy was employed to probe the “model” reaction mixture containing [CoN_5_^bpy^]^2+^, 4CzIPN, and TEOA under irradiation with visible light. As shown in Figure 6a, the preirradiation spectrum of the solution displayed peaks at 504 and 554 nm corresponding to [CoN_5_^bpy^]^2+^ and at 433 nm for 4CzIPN, suggesting no electron transfer in the dark. Under irradiation, the color of the CO_2_-saturated reaction mixture changed from yellow to dark brown with the appearance of new absorbance bands at 420 and 550 nm (Figure 6b). The intensity of the bands gradually increased with the exposure time and plateaued at ∼30 min, fitting a sigmoidal process (Figure 6c). In contrast, when 4CzIPN was irradiated in the presence of TEOA, a decrease in absorbance was observed for the characteristic bands at 363 and 432 nm with a concomitant increase at 319 and 328 nm (Figure S19), consistent with the reductive quenching of 4CzIPN* by TEOA. This indicates that the new absorption bands observed during photocatalysis at 420 and 550 nm can be attributed to the reduced [CoN_5_^bpy^] species. Upon removing the light source, the absorption spectrum of the photolysis solution returned to the preirradiation state, albeit with an additional band at ∼630 nm, suggesting that the catalyst did not undergo photodegradation (Figure 6b—inset). The additional peak at 630 nm could be related to coordination of a different ligand to Co in the sixth position when the catalyst returned to its resting state, [CoN_5_^bpy^]^2+^. The spectral change during photocatalysis was corroborated by spectroelectrochemical (SEC) analysis of the CoN_5_^bpy^ dimer (Figure 6d). Electrochemically reduced [CoN_5_^bpy^] solution at −2.0 V exhibited two absorption bands at 418 and 555 nm, and reoxidation of [CoN_5_^bpy^] at +0.2 V regenerated the original spectrum with an additional band at 630 nm. The SEC data are consistent with the spectral changes observed during photocatalysis, indicating that the electron transfer from [4CzIPN]^•–^ to [CoN_5_^bpy^]^2+^ generated the reduced Co species to initiate CO_2_ binding and its subsequent photoreduction to CO.

**Scheme 1:**
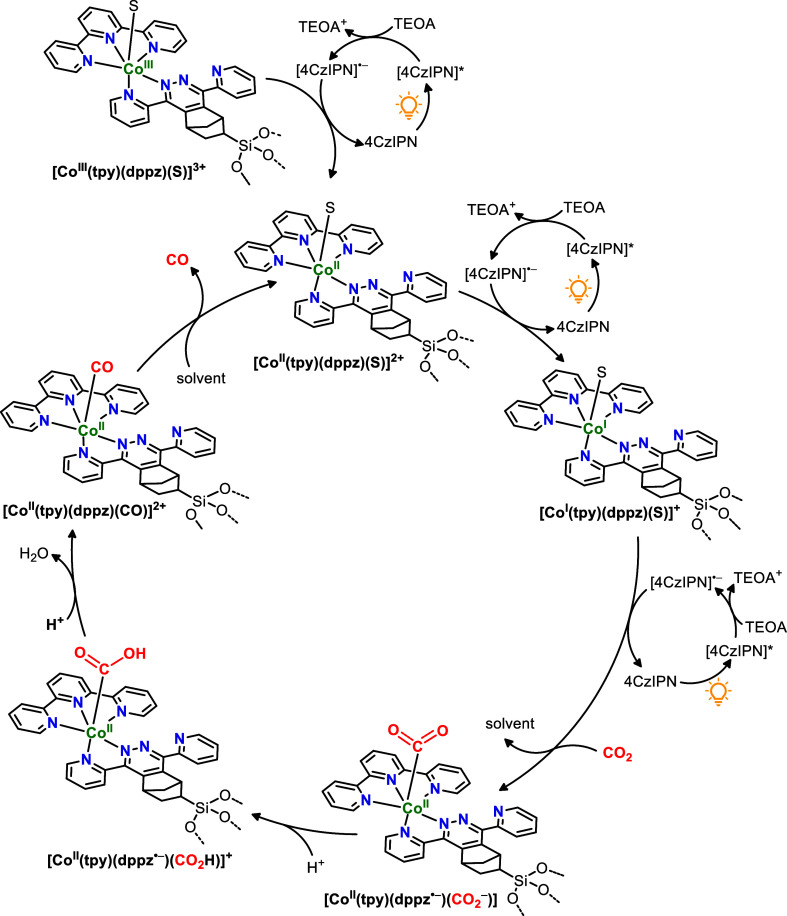
Mechanistic Proposal for Photocatalytic CO_2_ Reduction Cycle Based on CoN_5_ Heterogeneous Catalytic Sites

Based on literature reports,^5,54,66−68^ and supported by our mechanistic investigations, a tentative photocatalytic cycle for the CoN_5_-PMO/4CzIPN/TEOA photosystem is proposed in Scheme 1. The photoexcited 4CzIPN* is reductively quenched by TEOA to form [4CzIPN]^•–^, which has sufficient reducing power to reduce the [CoN_5_]-active sites in PMO and initiate the CO_2_ reduction catalytic cycle. The [CoN_5_] center undergoes two metal-centered reduction by [4CzIPN]^•–^, and a further ligand-centered reduction to yield [Co^I^(tpy)(dppz^•–^)(S)] species anchored to PMO (S = solvent). We hypothesize that partial delocalization of the electron within the dppz ligand stabilizes the reduced species and prevents its degradation. Subsequent dissociation of the solvent and binding of CO_2_ forms an intermediate species, [Co^II^(tpy)(dppz^•–^)(CO_2_^–^)]. Protonation of this intermediate by TEOA leads to the formation of [Co^II^(tpy)(dppz^•–^)(CO_2_H)]^+^ which is subsequently converted to [Co^II^(tpy)(dppz)(CO)]^2+^ through C–O bond cleavage and the release of water. Finally, the catalyst is regenerated by the dissociation of the Co–CO bond and release of CO, restoring [Co^II^(tpy)(dppz)(S)] species. We speculate that the slightly lower activity observed during the first cycle of recycling tests could be related to the hypothesis that CoN_5_-PMO serves as a precatalyst and requires a reduction step to form the active Co^II^ catalyst. Furthermore, use of 5 times higher catalyst loading in the recycling experiment could also contribute to a slow reduction process as light scattering in a concentrated suspension becomes a limiting factor.

## Conclusions

In summary, we have successfully demonstrated modulation of coordination structures of heterogenized molecular catalysts to control CO_2_ photoreduction activity in colloidal suspension. A dppz-functionalized PMO was used as a scaffold for immobilization of [M(tpy)] and [M(bpy)] moieties (M = Co or Ni) to construct four different materials consisting of Co- or Ni-active sites with different numbers of coordinated N atoms coordinated with the metal center. Among the series, CoN_5_-PMO displayed superior activity toward photoreduction of CO_2_, demonstrating that both the nature of metal center and the primary coordination structure strongly influence the catalytic performance. The precious metal-free photosystem containing CoN_5_-PMO reached a TON_CO_ value of 92 after 3 h at ∼75% CO selectivity, and the deactivation of the system was caused by the photodegradation of the sensitizer. Recycling experiments demonstrated that the CO evolution remained nearly constant over six cycles with an average TOF_CO_ value of 19.3 h^–1^. To the best of our knowledge, this work presents a rare example of utilizing mesoporous organosilica-based catalysts in a precious metal-free photosystem for CO_2_ reduction. Notably, the heterogeneous catalyst (CoN_5_-PMO) outperformed the analogous molecular Co complex (CoN_5_^dppz^) in solution, highlighting the advantage of catalyst immobilization. The coordination structure of the Co-active site in CoN_5_-PMO was confirmed by XAS analysis. Electrochemical studies on CoN_5_^dppz^ under homogeneous conditions indicated that the dppz ligand likely plays an important role in catalysis by enabling the reduction of the active site at milder potential. This work develops a versatile strategy for regulating the molecular structure of catalytic metal sites grafted on a mesoporous support and highlights the interplay between the coordination environment and catalytic CO_2_ reduction.

## Experimental Section

### Synthesis of Materials

#### [{Co(tpy)(bpy)}_2_(μ-O_2_)](PF_6_)_4_] (CoN_5_^bpy^ Dimer)

The cobalt dimer was synthesized by using a slightly modified reported method.^48^ A methanolic solution of Co(tpy)Cl_2_ (120 mg, 0.33 mmol), 2,2′-bipyridine (55 mg, 0.35 mmol), and NH_4_PF_6_ (130 mg, 0.78 mmol) was stirred at room temperature for 10 min. The resulting precipitate was collected by filtration and washed with methanol (2 × 10 mL) and diethyl ether. The isolated solid was recrystallized from acetone/diethyl ether, yielding 131 mg (53%) product as a brown powder.

#### [Co(tpy)(dppz)Cl](PF_6_)_2_

A mixture of Co(tpy)Cl_2_ (155 mg, 0.42 mmol) and the dppz ligand (100 mg, 0.42 mmol) was stirred in ethanol (30 mL) for 3 h to yield a pale brown solution. A solution of iodine (130 mg, 0.52 mmol) in ethanol (6 mL) was added dropwise to the resulting solution with stirring. After stirring for 10 min, the mixture was filtered. The brown filtrate was concentrated under reduced pressure to ∼2 mL, followed by the addition of a methanolic solution of NH_4_PF_6_ (500 mg, 3.1 mmol, 3 mL). The precipitated brown solid was collected by filtration to give 219 mg of product (59% yield). ESI-MS (positive mode): [M–1PF_6_^–^]^+^ = 706.0503 (706.0521), [M–2PF_6_^–^]^2+^ = 280.5426 (280.5434), [M–2PF_6_^–^–Cl^–^]^3+^ = 175. 3746 (175.3725). ^1^H NMR (DMSO-*d*_6_): δ (ppm) = 10.09 (d, *J* = 5.4 Hz, 1H), 9.39 (d, *J* = 8.0 Hz, 1H), 9.28 (m, 2H), 9.20 (d, *J* = 8.9 Hz, 1H), 9.16 (d, *J* = 7.5 Hz, 2H), 9.09 (dd, *J* = 8.8, 7.0 Hz, 1H), 9.00 (t, *J* = 7.3 Hz, 1H), 8.95 (d, *J* = 7.5 Hz, 1H), 8.90 (d, *J* = 8.9 Hz, 1H), 8.86 (d, *J* = 7.7 Hz, 1H), 8.72 (d, *J* = 4.2 Hz, 1H), 8.60 (t, *J* = 6.6 Hz, 1H), 8.36 (t, *J* = 7.7 Hz, 3H), 7.94 (td, *J* = 7.8, 1.5 Hz, 1H), 7.64–7.59 (m, 3H), 7.49 (dd, *J* = 9.8, 3.6 Hz, 1H), 7.44 (d, *J* = 5.4 Hz, 1H), 7.14 (d, *J* = 7.9 Hz, 1H). ^13^C NMR (DMSO-*d*_6_): δ 161.76, 161.54, 156.53, 156.43, 156.29, 156.19, 154.86, 154.35, 153.46, 152.75, 150.76, 150.11, 145.73, 144.12, 143.37, 142.57, 138.40, 132.30, 132.11, 131.21, 130.45, 130.10, 128.39, 127.50, 127.23, 126.57, 126.01, 121.53. ^19^F NMR (DMSO-*d*_6_): δ (ppm) = −70.1 (d, *J*_P–F_ = 712 Hz). FTIR (ATR), ν (cm^–1^) 1608, 1573, 1484, 1455, 1423, 1333, 1247, 1204, 1171, 1142, 1100, 1035, 827, 769, 555. UV–vis, λ_max_, nm (ε, M^–1^ cm^–1^): 447 (366 M^–1^ cm^–1^), 507 (shoulder, 226 M^–1^ cm^–1^), 553 (weak shoulder).

#### CoN_5_-PMO

NdppzPMO (30 mg) was suspended in a methanolic solution (10 mL) of Co(tpy)Cl_2_ (6 mg, 0.017 mmol), and the mixture was refluxed overnight under a nitrogen atmosphere. The resulting solid was collected by filtration, washed with methanol to remove any unreacted Co(tpy)Cl_2_ complex, and dried under vacuum to give CoN_5_-PMO (30 mg).

CoN_4_-PMO, NiN_4_-PMO, and NiN_5_-PMO were prepared according to the above-described method using Co(bpy)Cl_2_, Ni(bpy)Cl_2_, and Ni(tpy)Cl_2_, respectively, instead of Co(tpy)Cl_2_.

#### CoN_2_-PMO

NdppzPMO (30 mg) was suspended in a THF solution (10 mL) of CoCl_2_·6H_2_O (4 mg, 0.017 mmol), and the mixture was refluxed overnight under a nitrogen atmosphere. The resulting solid was collected by filtration, washed with THF and methanol to remove any unreacted CoCl_2_·6H_2_O, and dried under vacuum to give CoN_2_-PMO (30 mg). Co loading from ICP: 0.324 mmol of Co g^–1^.

### Photocatalytic CO_2_ Reduction

Photocatalytic CO_2_ reduction experiments were performed in 10 mL clear glass screw vials (Thermo Fisher, catalogue number 10-SV) sealed with rubber septa. In a typical photocatalytic reaction, PMO catalyst (1 mg) was dispersed in 9:1 (v/v) MeCN/TEOA (4 mL) mixture containing 0.5 mM photosensitizer (Ru(bpy)_3_^2+^ or 4CzIPN). For selected samples, 10 mM BIH was added to the reaction mixture as an electron donor. The mixture was purged with CO_2_ for 15 min and irradiated under 1 sun illumination (100 mW cm^–2^) using a SciSun-LP-150 solar simulator. For the homogeneous counterpart experiments, the molecular catalyst (0.04 μmol) was added to the reaction mixture (4 mL) instead of the heterogeneous catalyst, followed by purging with CO_2_. Control experiments were conducted under similar conditions by suppressing one component of the photocatalytic system (*i.e.*, visible light, heterogeneous catalyst, photosensitizer, sacrificial electron donor, and CO_2_) to assess the influence of each parameter individually. The temperature of the photocatalysis mixture was maintained at ∼27 °C.

The catalytic activity was expressed in terms of TON_CO_, determined as moles of CO produced per mole of cobalt or nickel, while the selectivity of the system toward CO was estimated according to following equation:
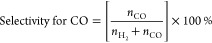


#### Recycling Experiments

The PMO catalyst (5 mg) was subjected to multiple photocatalytic cycles of 1 h irradiation under identical conditions. After each photocatalytic CO_2_ reduction cycle, the catalyst was collected by centrifugation and washed three times with acetonitrile to remove physisorbed species. The resulting solid was dried under vacuum and used for subsequent recycling experiments by adding fresh photosensitizer and MeCN/TEOA solution.

## Supporting Information Available

Experimental methods and instrumentation, additional data on materials characterization, electrochemical analysis, and CO_2_ photoreduction including quantum yield calculations, photoluminescence tests, and *in situ* UV–vis characterization (PDF)
