# Supplementary material for: Manipulating the Coordination Structure of Molecular Cobalt Sites in Periodic Mesoporous Organosilica for CO2 Photoreduction
Source: ACS Appl Energy Mater. 2024 Jun 29;7(14):5924–36. doi: 10.1021/acsaem.4c01161 (PMC11267497; doi:10.1021/acsaem.4c01161)
Supplement: Supplementary file 1 — ae4c01161_si_001.pdf [file ae4c01161_si_001.pdf]

Supporting Information

Manipulating the Coordination Structure of Molecular Cobalt Sites in Periodic Mesoporous Organosilica for CO_2 Photoreduction

Raúl Rojas-Luna,[a,b] Francisco J. Romero-Salguero,[a] Dolores Esquivel,*[a] Souvik Roy*[b]

[a]*Departamento de Química Orgánica, Instituto Químico para la Energía y el Medioambiente (IQUEMA), Facultad de Ciencias, Universidad de Córdoba, Campus de Rabanales, Edificio Marie Curie, E-14071 Córdoba, Spain.*
[b]*School of Chemistry, University of Lincoln, Green Lane, Lincoln, LN6 7DL, UK*

Email: q12esmem@uco.es (Dolores Esquivel), sroy@lincoln.ac.uk (Souvik Roy)

Table of Contents

1. Experimental Section 2
2. Supporting Figures 6
3. Supporting Tables 20
4. References 23

1. Experimental Section

Chemicals and materials. All organic synthesis reactions were carried out under inert atmosphere using standard high-vacuum and Schlenk techniques unless otherwise noted. Reagents and solvents, including dry solvents, were purchased from commercial suppliers and used as received. Cobalt(II) chloride hexahydrate (Acros Organics, 98%), nickel(II) chloride hexahydrate (SigmaAldrich, 98%), 2,2':6',2"-terpyridine (BLDpharm, 99.98%), 2,2′-bipyridyl (Fluorochem, 98%), ammonium hexafluorophosphate (NH_4PF_6, Fluorochem, 99%), triethanolamine (Sigma-Aldrich, ≥99%), acetonitrile (MeCN, Fischer, ≥99.8%), methanol (MeOH, Fischer, ≥99.9%), methanol extra dry (Acros Organics, 99.8%), tetrahydrofuran anhydrous (THF, Sigma, ≥99.9%) ethanol absolute (EtOH, Fischer, ≥99.8%), diethyl ether (Fischer, ≥99,5%), 2-phenylbenzimidazole (Sigma, 97%), iodomethane (Sigma, 99%), sodium borohydride (Sigma, ≥98%), tris(2,2′-bipyridyl)dichlororuthenium(II) hexahydrate (Sigma, 100%), purpurin (Sigma, 90%) 9-cyanoanthrachene (TCI, ≥95%) and methanesulfonylmethane (MSM, Fluorochem, 99 %) were used as received without further purification. NdppzPMO,[1] Co(bpy)Cl_2,[2] Ni(bpy)Cl_2,[3] Co(tpy)Cl_2,[4] Ni(tpy)Cl_2,[4] dppz (3,6-dipyridin-2-ylpyridazine),[5] [Ru(bpy)$_3$](PF_6)$_2$,[6] BIH,[7] 4CzIPN,[8] and etPMO[9,10] were prepared according to synthetic procedures reported in literature. Ni(tpy)Cl_2 preparation was adapted by using EtOH instead of THF. It should be noted that the dppz abbreviation used in the manuscript presents 3,6-dipyridin-2-ylpyridazine molecule and not be confused with dipyridophenazine structure which is also often abbreviated as dppz in the literature.

Characterization techniques and methods. X-ray diffraction patterns (XRD) were acquired on a Bruker D8 Discover A25 diffractometer equipped with a monochromatic Cu-Kα radiation (40 kV and 30 mA, $\lambda = 1.5418$ Å) in the 2θ range of 0.5-5°. Nitrogen adsorption/desorption isotherms were performed at liquid nitrogen temperature (−196 °C) using a Micromeritics ASAP 2420 instrument after degassing the sample at 120 °C overnight. Multipoint Brunauer−Emmett−Teller (BET) method was employed for the estimation of the specific surface area (S_{BET}). Structural analyses were accomplished by TEM using a JEOL JEM 1400 microscope at an acceleration voltage of 300 kV. Raman measurements were performed using a Bruker Senterra benchtop Raman microscope using 785 nm laser excitation and a 50× objective lens. The spectrometer was calibrated

using a polystyrene standard. Raman spectra were collected over the 50-2630 cm^{-1} spectral range at 25 mW power with 40 s exposure time and 200 accumulations. UV-Vis spectra were acquired on a Shimadzu UV-1800 Spectrophotometer using quartz glass cuvettes (1 cm path length). UV-Vis DRS measurements were recorded on a Perkin Elmer Lambda 650 S UV/Vis spectrometer, which operates in a double beam mode with a 150 mm integrating sphere. X-ray photoelectron spectroscopy (XPS) was performed using a Thermo Fisher Scientific K-alpha+ spectrometer fitted with a micro-focused monochromatic Al Kα X-ray source. Data were recorded at pass energies of 150 eV for survey scans and 40 eV for a high-resolution scan with 1 eV and 0.1 eV step sizes, respectively, at a spot size of approximately 200 µm. C 1 s electron at 284.8 eV was used as a standard reference to calibrate the photoelectron energy shift. All the data analysis was performed on the Casa XPS software. ^{1}H NMR spectra were collected on a Bruker AVANCE III HD spectrometer using 500 MHz. CHN elemental analysis were determined in an elemental microanalyzer Thermo Finnigan Flash EA 1112 series with a Micro TruSpec detection system from LECO. ICP analysis was performed on a Thermo Scientific iCAP 7000 series ICP-OES Spectrometer, using the Qutegra software. Calibration was performed in the range of 0.1-10 ppm. Each sample was measured with three replications. The steady state photoluminescence spectra were collected on a FLS980 fluorescence spectrometer. Thermal analysis was performed on Netzsch STA 449 F3 model attached with mass spectrometer QMS403D Aeolos. During the measurements, the purge gas flow (N_2) and protective gas flow (N_2) were 50 mL min^{-1} and 20 ml min^{-1}, respectively. The samples were heated from 30 °C to 700 °C at a rate of 10 °C min^{-1} with simultaneous mass spec analysis of the evolved gas. XAS data was collected at beamline B18 at the Diamond Light Source (UK Catalysis Hub, Block Allocation Group, SP29271-8). Pellets (13 mm) were prepared by homogeneously missing the samples with an inert cellulose matrix and mounted on a sample holder using Kapton tape. XAS measurements were performed in the transmission mode for $CoCl_2$, Co(bpy)Cl_2, and CoN_5-dimer (3 scans), while CoN_5-PMO and CoN_4-PMO were measured in fluorescence mode (6 scans). XAS data processing and EXAFS analysis were performed using Athena and Artemis software, respectively. Cyclic voltammetry of CoN_5-dimer was performed on a Biologic VSP-300 potentiostat using a conventional three electrode set up with a glassy carbon working electrode, a Pt-wire counter electrode, and a non-aqueous Ag|Ag^+ (10 mM $AgNO_3$ in MeCN electrolyte) reference electrode. A 0.1 M solution of Bu_4NPF_6 in neat MeCN or

MECN/TEOA mixture (9:1) was used as supporting electrolyte. The voltammograms were referenced by the addition of ferrocene as an internal standard after the final experiment, and all potentials given in this work are reported against the $Fc^{+/0}$ couple.

Photocatalysis experiments were performed using a SciSun-LP-150 solar simulator from ScienceTech, equipped with an air mass 1.5 global filter (AM 1.5G) and a UV-filter (>400 nm). The gas phase photolysis products (H_2 and CO) were quantified by gas chromatography (SRI 8610C) analysing 50 μL samples of the vessel headspace along the reaction time. The CO and H_2 peaks were determined by calibration with standard CO and H_2 gas, whilst liquid phase was analysed by ^{1}H NMR and ion chromatography. The SRI gas chromatograph (multiple gas analyser #1) is equipped with a thermal conductivity detector (TCD) and a flame ionisation detector (FID) with a built-in methaniser attachment. A silica gel column (6 ft) column was used to block CO_2 and H_2O, and molecular sieve 13X (6 ft) main column was used to separate H_2 and CO. N_2 was used as the carrier gas at 23 psi pressure.

For HCOOH quantification, 1 M KOH (0.1 mL) was added to the photoirradiated suspension, following by solvent evaporation under reduced pressure. The residue was extracted with D_2O (1 mL) and filtered to remove the insoluble species. Then, a known amount of MSM (typically 5-6 mg) was added as internal standard. Finally, the solution is filtered and transferred into a NMR tube for HCOOH quantification by ^{1}H NMR spectroscopy.

Quantum yield determination. Apparent quantum yield (AQY) for CO_2 photoreduction was calculated according to the following equation:

$$\mathrm{AQY}(\%) = \frac{\mathrm{CO\ evolution\ rate\ (mol\ s^{-1})}}{\mathrm{photon\ flux\ rate\ (Einstein\ s^{-1})}}\ \mathrm{x}\ 100$$

The quantum yield was measured under similar photocatalytic reaction conditions. Determination of the CO evolution rate was accomplished by irradiating with a monochromatic Blue LED (467 nm) a CO_2 saturated suspension of CoN_5-PMO (2 mg) in 4 mL MeCN/TEOA (9:1 v/v) containing 4CzIPN photosensitiser (0.5 mM). The slope of the linear fit in the region which the CO evolved increased linearly in the time dependent CO evolution curve gave a CO evolution rate value of 8.02×10^{-10} mol s^{-1} (Figure S13**)**. The photon flow rate was determined by using $K_3Fe(C_2O_4)_3$ as a standard

chemical actinometer following a reported procedure.[11,12] According to the actinometric method, the photon flux rate value was estimated as 1.577 $\times 10^{-7}$ Einstein s^{-1} (Figure S14).

Fluorescence quenching experiments. The quenching experiments were performed in 3 mL CH_3CN solution containing 40 μM 4CzIPN with different concentrations quencher (TEOA or CoN_5-dimer). The solution was excited at 400 nm, and the relative emission lifetime of the characteristic emission of the 4CzIPN organic sensitizer at 560 nm was recorded. The bimolecular quenching rate constant (k_q) was determined according to the Stern-Volmer equation.

$$\frac{I_0}{I} = 1 + K_{SV} = 1 + k_q\ \tau_0\ [Q]$$

Where I_0 and I are the emission intensity in the absence and presence of quencher, respectively, K_{SV} is the Stern-Volmer constant corresponding to the slope of the linear fit (k_q τ_0), k_q is the bimolecular quenching rate constant, τ_0 the excited state lifetime in the absence of quencher, and [Q] is the molar concentration of the quencher.

2. Supporting Figures

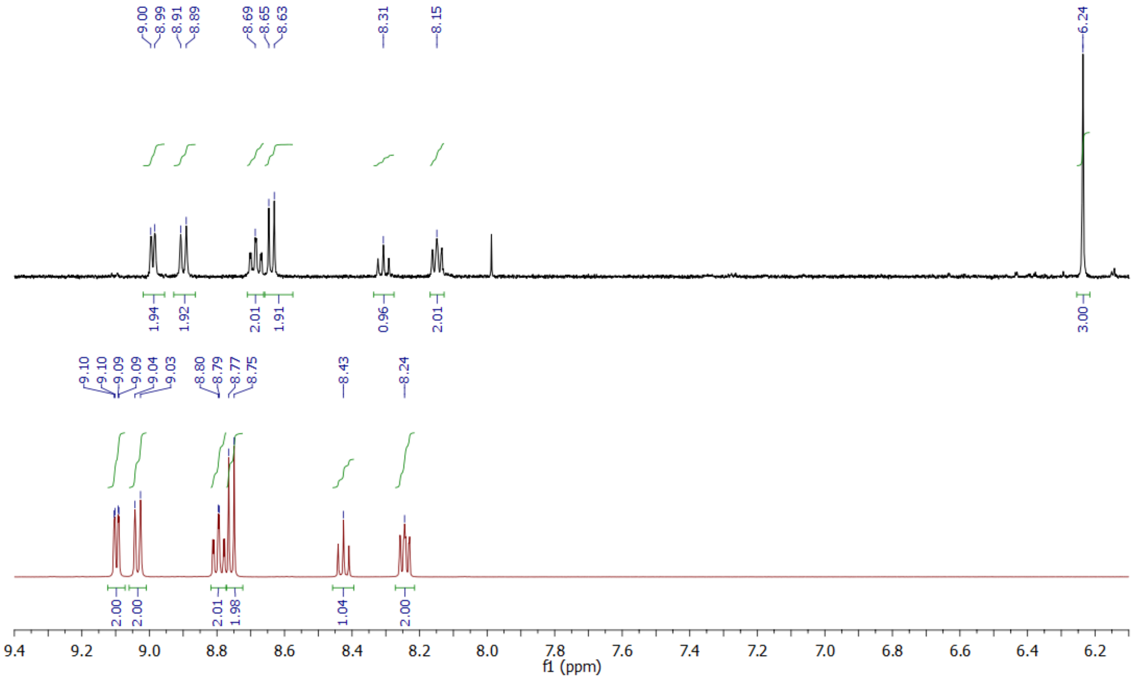

Figure S1. ^{1}H NMR spectra of digested CoN$_5$-PMO (black) and 2,2':6,2"-terpyridine in D_2SO_4/DMSO-d$_6$ mixture (1:6 v/v) at 293 K. The NMR sample was prepared by digesting 11 mg CoN$_5$-PMO in D_2SO_4/DMSO-d$_6$ mixture (700 µl) by mild heating (~50 °C) and ultrasonication, followed by syringe filtration (0.22 µm Nylon). During digestion, 1,3,5-trimethoxybenzene (5.3 mg, 31.5 µmol) was added as an internal standard, which corresponds to the 6.24 ppm peak in the top NMR spectrum.

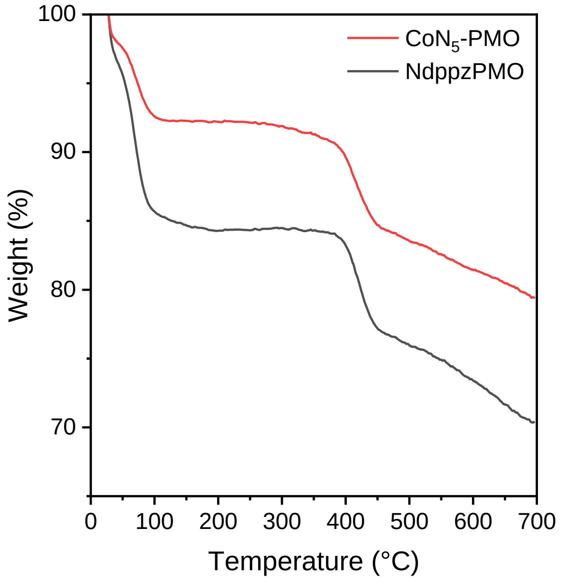

Figure S2. Thermogravimetric analysis (TGA) of CoN_5-PMO and NdppzPMO. The samples were heated from 30 °C to 700 °C under N_2.

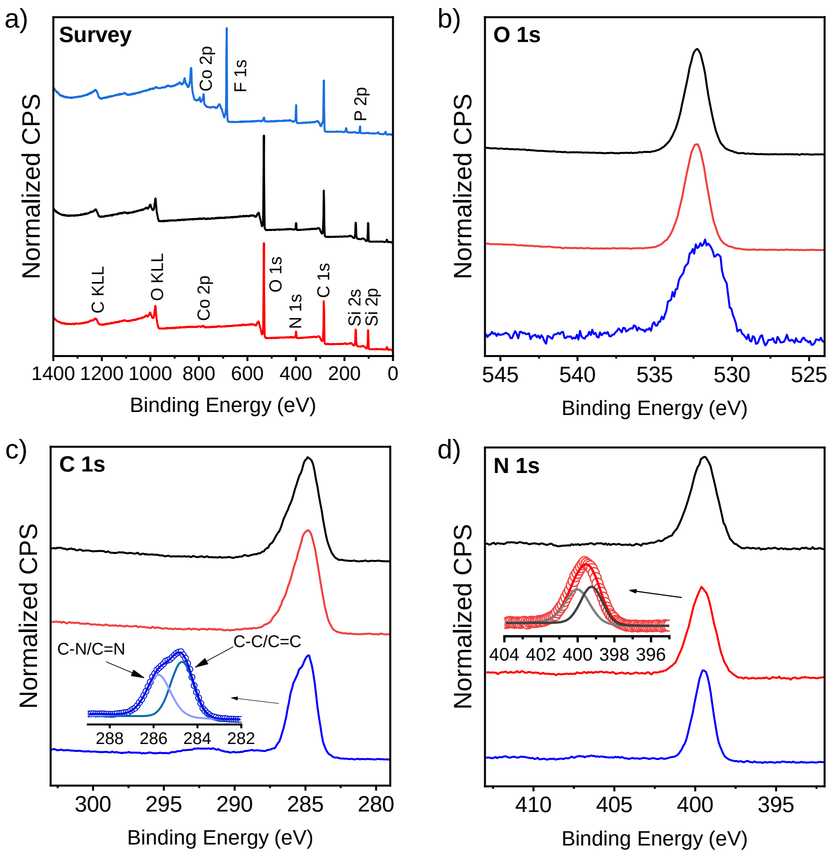

Figure S3. (a) Survey spectra, (b) O 1s, (c) C 1s and (d) N 1s XPS spectra of CoN_5-PMO pre-catalysis (black trace), CoN_5-PMO post-catalysis (red trace) and CoN_5^{bpy} (blue trace).

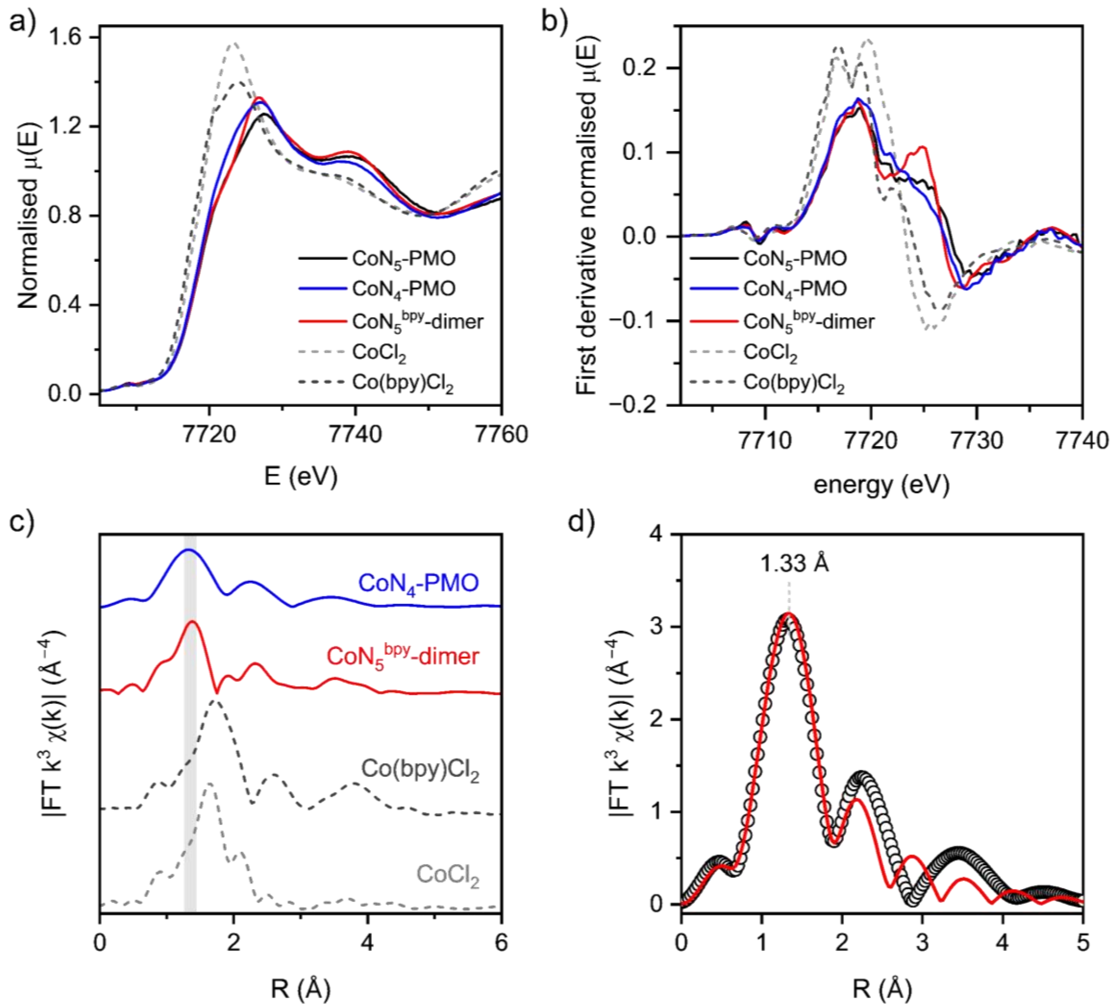

Figure S4. X-ray absorption data for CoN_5-PMO, CoN_4-PMO and the reference compounds. a) Co K-edge XANES spectra; (b) first derivative of the Co-K edge XANES data; (c) Fourier transformed R-space EXAFS data for CoN_4-PMO, CoN_5^{bpy}-dimer, Co(bpy)Cl_2 and $CoCl_2$ (shaded region shows shorter radial distance for the scattering paths corresponding to first coordination shell of CoN_4-PMO and CoN_5^{bpy}-dimer); (c) R-space EXAFS fitting for the first coordination shell of CoN_4-PMO with the data shown as open circles and the FEFF fit as solid red line.

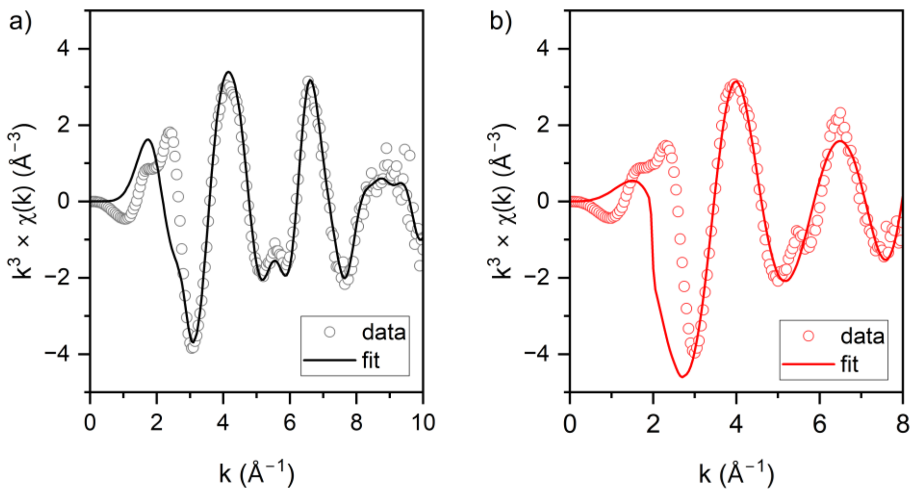

Figure S5. The EXAFS data in the k-space for a) CoN_5-PMO and b) CoN_4-PMO. The open circles and the solid line present the data and the fit, respectively. For CoN_4-PMO, only the first coordination shell was fitted.

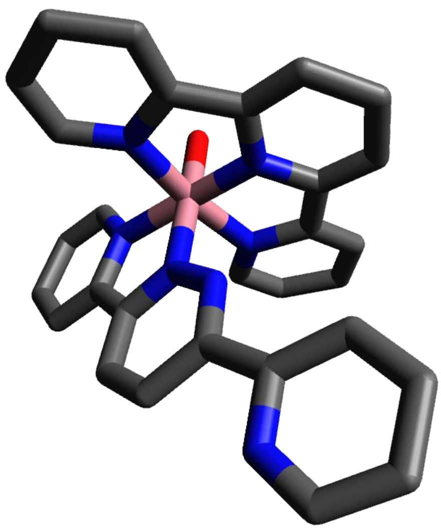

Figure S6. Structural model of Co(tpy)(dppz)(OH) based on the Co-N/O bond distances obtained from EXAFS fit (first coordination shell; Co – pink, C – grey, N – blue, O – red).

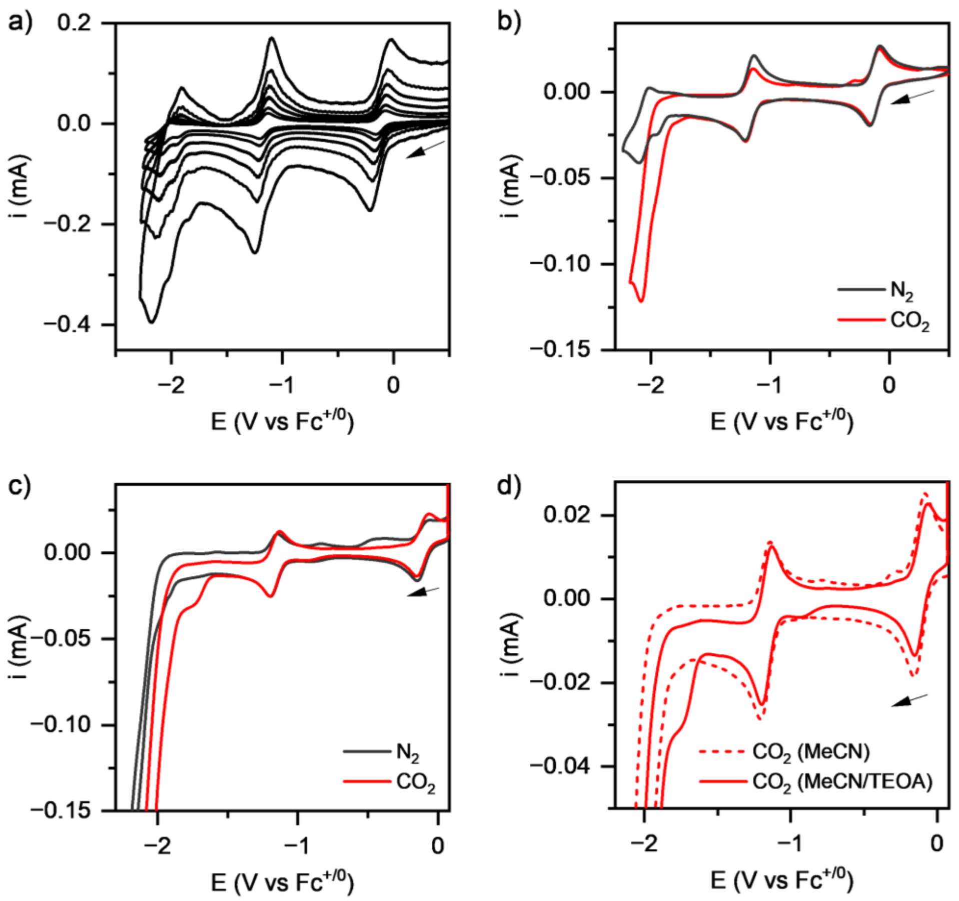

Figure S7. Cyclic voltammograms of CoN_5^{bpy} (1.5 mM) in MeCN (Figure a and b) and 9:1 MECN/TEOA mixture (Figure c). (a) CVs of CoN_5^{bpy} recorded at different scan rates (5, 2, 1, 0.5, 0.2, 0.1 V s^{-1}). (b) Catalytic CVs showing current enhancement under CO_2 saturated condition in MeCN. (c) Catalytic CVs showing current enhancement under CO_2 saturated condition in MeCN/TEOA. (d) Comparison on the CVs recorded under CO_2 saturation in neat MeCN and MeCN/TEOA mixture. Conditions: glassy carbon working electrode, Pt counter electrode, Ag/$AgNO_3$ (10 mM) reference electrode, and $^{n}Bu_4BF_4$ (0.1 M) supporting electrolyte

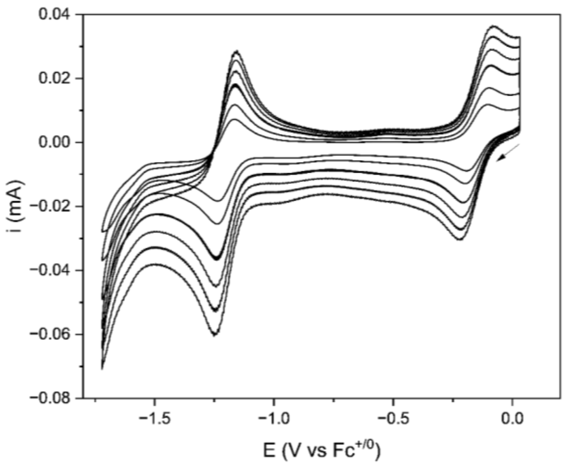

Figure S8. Cyclic voltammograms CoN_5^{dppz} (1.5 mM) in 9:1 MeCN/TEOA (v/v) under N_2 at various scan rates (500, 400, 300, 200, 100 and 50 mV s^{-1}). The black arrow indicates the scan direction. Conditions: glassy carbon working electrode, Pt counter electrode, Ag/$AgNO_3$ (10 mM) reference electrode, $^nBu_4BF_4$ (0.1 M) supporting electrolyte.

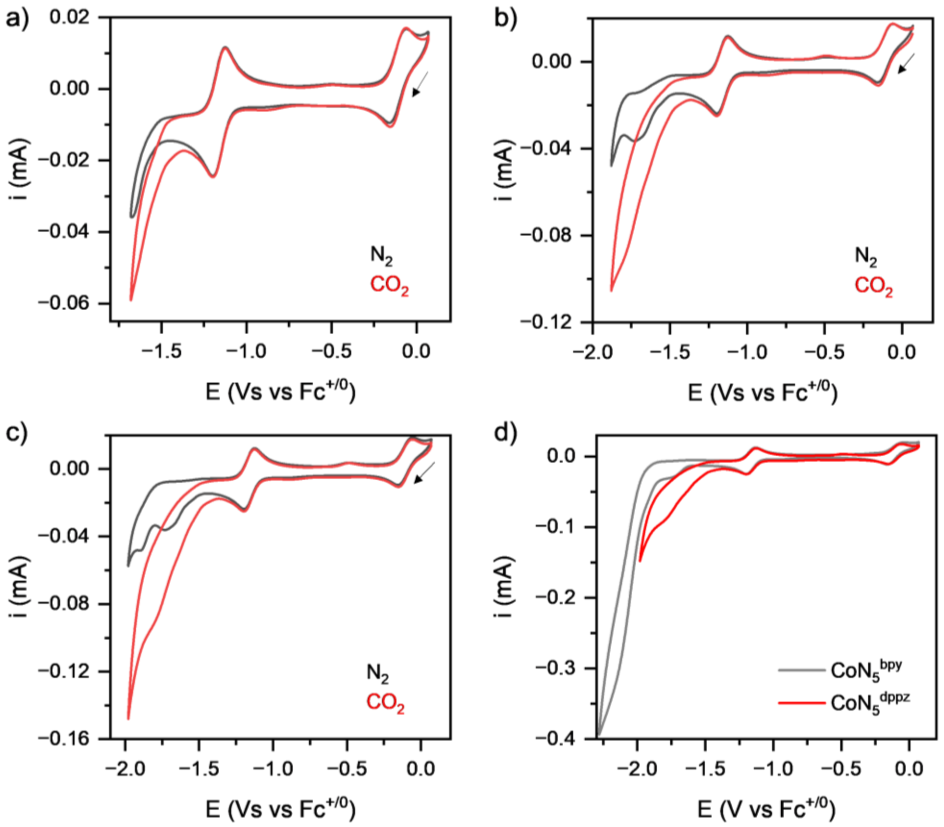

Figure S9. Cyclic voltammograms CoN_5^{dppz} (1.5 mM) in 9:1 MeCN/TEOA (v/v) under N_2 (black trace) and CO_2 (red trace) saturated conditions. Figure (a), (b) and (c) show CVs that were scanned over different potential ranges. Figure (d) compares the CVs of CoN_5^{dppz} and CoN_5^{bpy} recorded under CO_2 saturated conditions in MeCN/TEOA. The second scans are shown for all measurements. The black arrows indicate scan direction. Conditions: glassy carbon working electrode, Pt counter electrode, Ag/AgNO$_3$ (10 mM) reference electrode, $^nBu_4BF_4$ (0.1 M) supporting electrolyte, 100 mV s^{-1} scan rate.

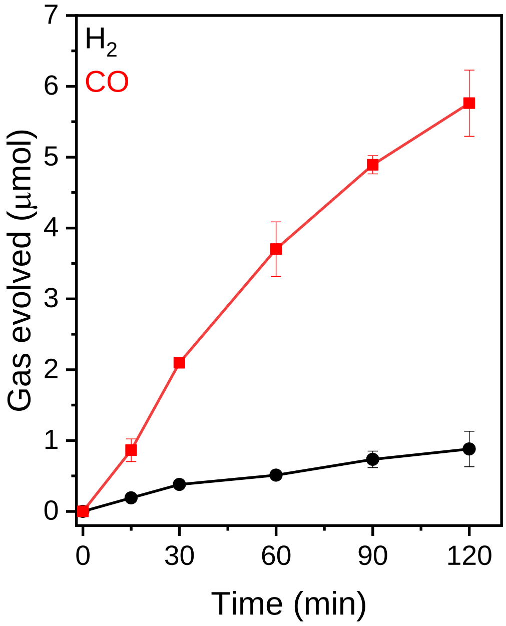

Figure S10. Time dependence of H_2 (black trace) evolution and CO evolution (red trace) and CO selectivity (red bar plot) for CoN_5-PMO (1 mg) in the presence of [Ru-PS] (0.5 mM) and BIH (10 mM) in 9:1 (v/v) MeCN/TEOA.

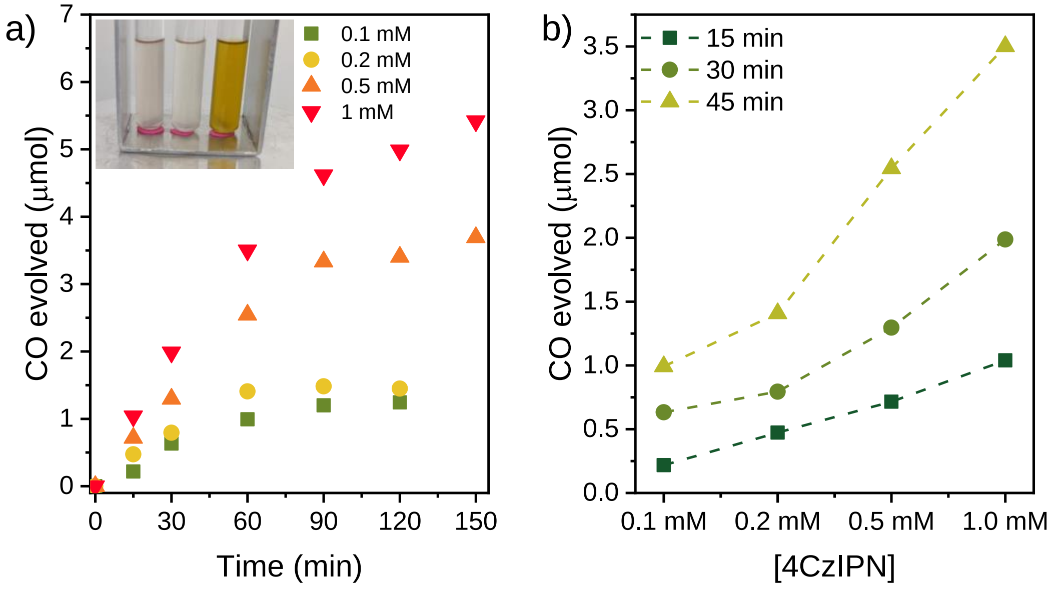

Figure S11. Influence of 4CzIPN concentration on photocatalytic CO generation in 4 mL CO_2 saturated MeCN/TEOA (10% v/v) solution containing 1 mg CoN_5-PMO during 1 h visible light (λ > 400 nm) irradiation. Inset image shows change in colour of the photocatalysis mixture with varying concentration of 4CzIPN (from left to right: 0.1, 0.2 and 1.0 mM) after 1 hour irradiation.

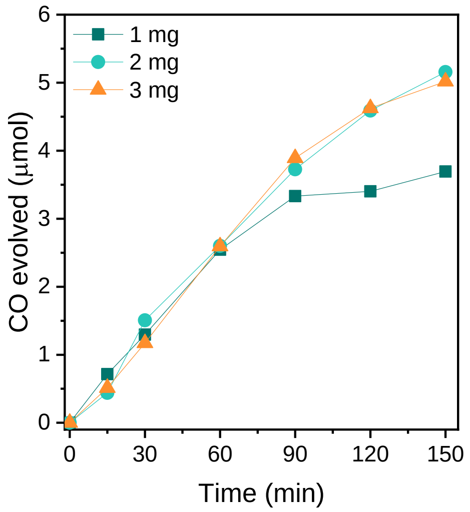

Figure S12. Effect of catalyst amount on photocatalytic CO generation in 4 mL CO_2 saturated MeCN/TEOA (10% v/v) solution containing 0.5 mM 4CzIPN during 1 h visible light (λ > 400 nm) irradiation.

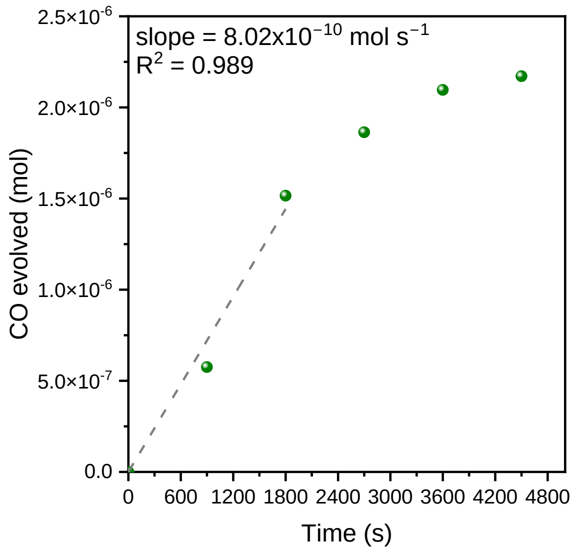

Figure S13. Time dependent CO evolution curve for CoN_5-PMO for the determination of the quantum yield in the photocatalytic CO_2 reduction at 467 nm monochromatic irradiation. Slope is plotted in the linear region of the curve (0-1800 s). Condition: CoN_5-PMO (2 mg) in 4 mL MeCN/TEOA (9:1 v/v) containing 4CzIPN photosensitiser (0.5 mM).

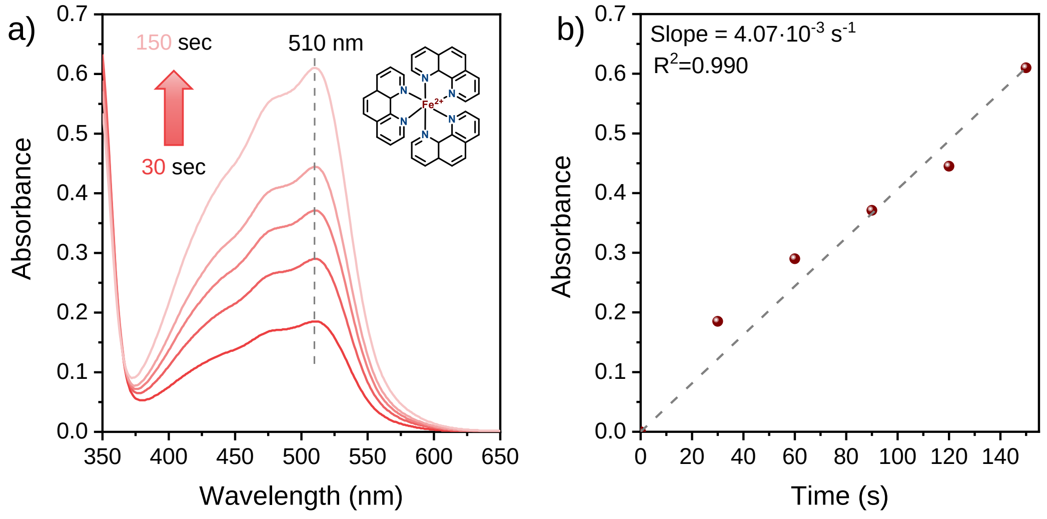

Figure S14. (a) UV-Vis absorption spectra changes at 30 seconds interval of the $[Fe(phen)_3]^{2+}$ complex obtained in the photoreduction of the $K_3Fe(C_2O_4)_3$ chemical actinometer by irradiation with a 467 nm Blue LED. (b) Absorption at 510 nm vs. irradiation time for $[Fe(phen)_3]^{2+}$ complex. The slope of the linear fit corresponds to the rate of Fe^{2+} species generated during the irradiation.

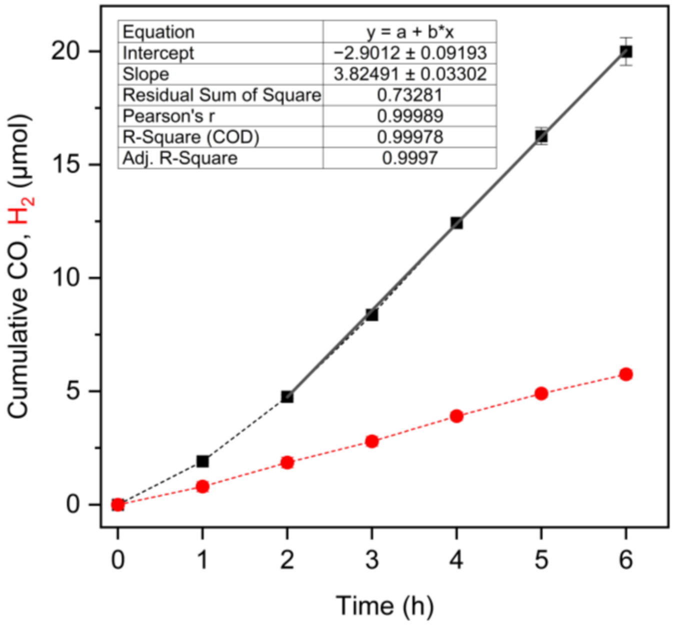

Figure S15. Linear fit of the cumulative CO evolution data from six one-hour recycling runs. The data for the first cycle was excluded from the fit because the CO evolution trace shows a clear induction period. The photocatalysis experiment was performed using 5 mg CoN_5-PMO in 4 mL MeCN/TEOA (9:1 v/v) containing 0.5 mM 4CzIPN.

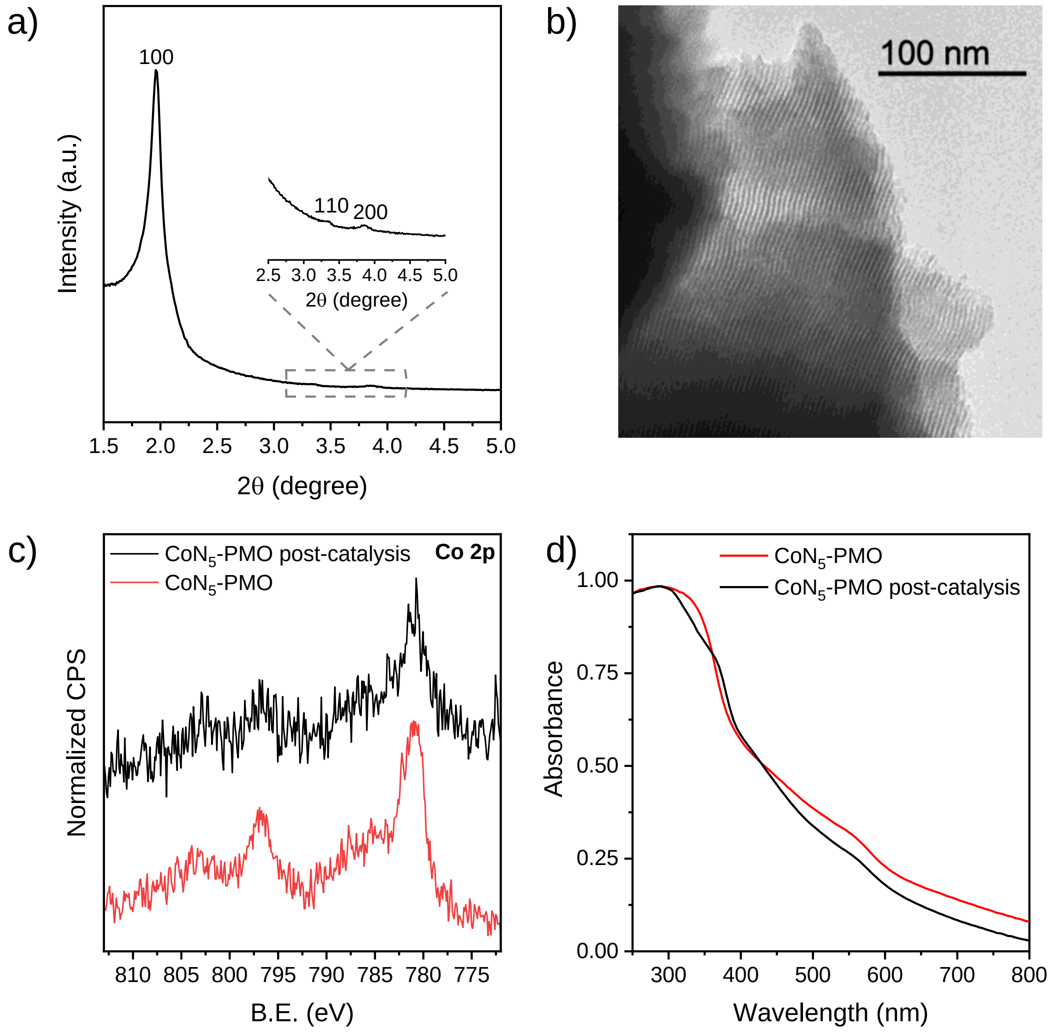

Figure S16. Characterization of CoN_5-PMO after six photocatalytic cycles: (a) Powder X-ray diffraction pattern; (b) TEM image; (c) UV-Vis diffuse reflectance spectrum and (d) Co 2p XPS spectrum.

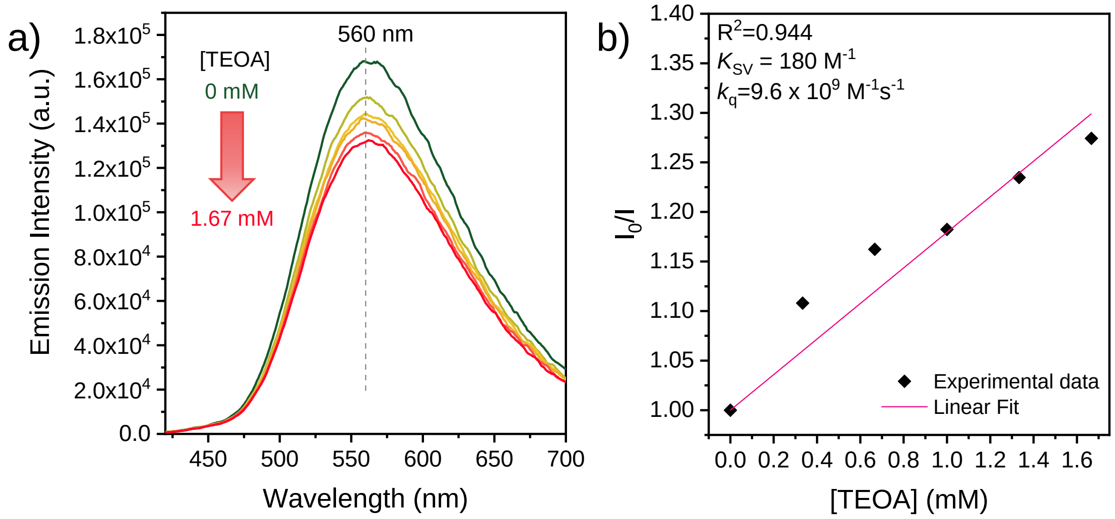

Figure S17. (a) Emission spectra of 4CzIPN* (40 μM) excited by monochromatic light at 400 nm in MeCN (3 mL) containing TEOA (0-1.67 mM). (b) Stern-Volmer plots of the emission quenching monitored at 560 nm.

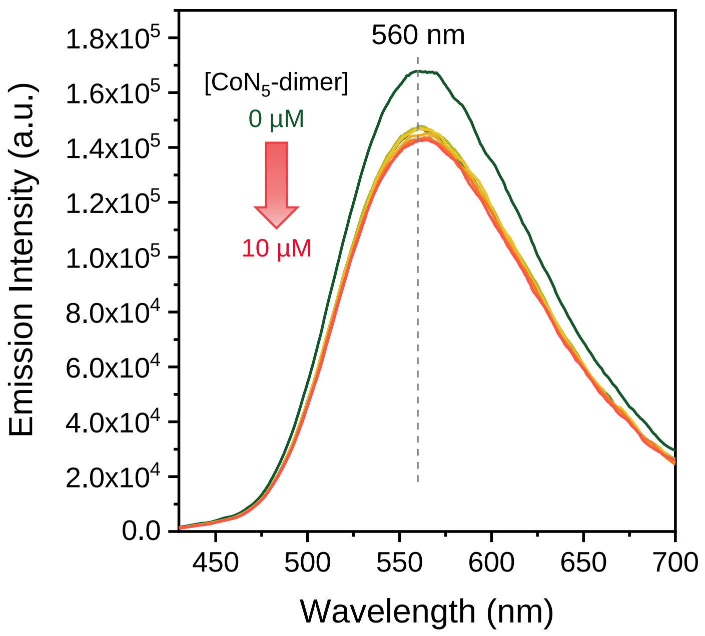

Figure S18. (a) Emission spectra of 4CzIPN* (40 μM) excited by monochromatic light at 400 nm in MeCN (3 mL) containing $[Co(tpy)(bpy)]_2O_2(PF_6)_4$ (0-10 μM).

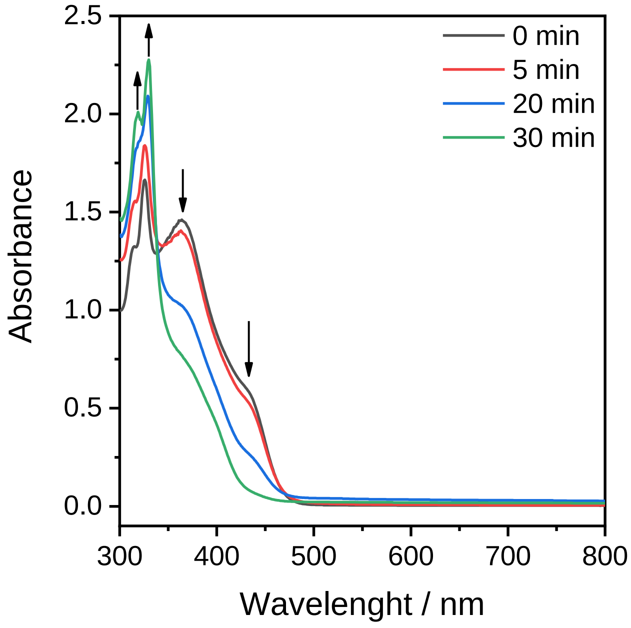

Figure S19. UV-Vis absorption spectra of 4CzIPN (0.1 mM) in CO_2-saturatated MeCN/TEOA (9:1 v/v).

3. Supporting Tables

Table S1. Metal loading of MN_x-PMO materials determined by ICP-OES analysis. The ICP samples were prepared by digesting ~3 mg solid in 1 mL conc. HNO_3 followed by dilution to 10 mL using deionised water.

**Sample**	**Weight (mg)**	**[Co] or [Ni] (ppm)**	**Co/Ni loading (mmol g^{-1})**
CoN_5-PMO	3	0.736	0.0415
CoN_4-PMO	3	1.236	0.0698
NiN_5-PMO	3.4	3.794	0.1692
NiN_4-PMO	3.8	3.745	0.1867
CoN_5-PMO (post-catalysis)	3	0.312	0.0176

Table S2. EXAFS fitting parameters for the first and second coordination shells of CoN_5-PMO, corresponding to Figure 3c in the manuscript. The FEFF input model was derived from the crystal structure of $[\{Co(tpy)(bpy)\}_2(\mu\text{-}O_2)\}](PF_6)_2$. To minimise over-parametrisation during EXAFS fit, the coordination numbers (N) were set from FEFF model and S_0^2 was set at 1 for all paths. The fitted value of ΔE_0 was –8.04 (all paths). The second shell (peak at R = 2.30 Å) was fitted using Co-C/N single-scattering paths (distance 2.75-3.00 Å), originating from the terpyridine and dppz ligands. The EXAFS peak at R = 3.31 Å originates from Co-C-C/N and Co-N-C/N multiple-scattering pathways (distance 4.10-4.70 Å, triangle scattering) from the terpyridine and dppz ligands.

Shell	Bond	Degeneracy (N)	R (Å)	$\sigma^2 \times 10^{-3}$ ($Å^2$)
1	Co-N	4	1.97	7.6
1	Co-N	1	1.80	7.1
1	Co-O	1	1.82	2
2	Co-C	6	2.86	9
2	Co-N	1	2.99	9
2	Co-C	1	3.08	9

Table S3. EXAFS fitting parameters for the first coordination shell of CoN_4-PMO, corresponding to Figure S4 in the manuscript. The FEFF input model was based on a structural model of [*cis*-Co(bpy)(dppz)$(OH_2)_2$]$^{3+}$ that was derived from the crystal structure of [*cis*-Co$(bpy)_2(OH_2)_2$]$(PF_6)_3$. The coordination numbers (N) were set from FEFF model and S_0^2 was set at 1 for all paths. The fitted value of ΔE_0 was –9.98 (all paths). R factor for the fit is 0.02178 and reduced chi-square value is 549.91.

Shell	Bond	Degeneracy (N)	R (Å)	$\sigma^2 \times 10^{-3}$ (Å^2)
1	Co-N	3	1.90	0.4
1	Co-N	1	1.99	5.7
1	Co-O	2	2.10	3.9

Table S4. Control experiments for CO_2 photocatalytic reduction.[a]

Entry	Photosensitiser	Catalyst	e^- donor	Product (µmol mg^{-1})	
				CO	H_2
1	[Ru-PS]	-	TEOA	0.22	0.75
2		-	BIH[b]	0.21	0.16
3		NdppzPMO	TEOA	0.20	0.78
4[c]		CoN_5-PMO	TEOA	-	-
5[d]		CoN_5-PMO	TEOA	-	0.91
6	4CzIPN	-	TEOA	-	-
7		-	BIH[b]	-	-
8		NdppzPMO	TEOA	-	0.20
9[c]		CoN_5-PMO	TEOA	-	-
10[d]		CoN_5-PMO	TEOA	-	6.23
11		$Co(tpy)Cl_2$[e]	TEOA	1.28	0.14
12		etPMO[f] + $Co(tpy)Cl_2$[e]	TEOA	1.25	0.40
13	-	CoN_5-PMO	TEOA	-	-

[a]Condition for standard photocatalysis experiments: 1 mg solid catalyst (CoN_5-PMO, NdppzPMO, etPMO or CoN_2-PMO), 4 mL MeCN/TEOA (9:1), 0.5 mM photosensitiser ($[Ru(bpy)_3]^{2+}$ or 4CzIPN), visible light (100 mW cm^2, AM 1.5G, $\lambda > 400$ nm), 1 h irradiation. [b]10 mM BIH in 9:1 MeCN/TEOA. [c]Dark. [d]Nitrogen saturation [e]0.041 µmol $Co(tpy)Cl_2$ in 4 mL MeCN/TEOA (9:1 v/v). [f]etPMO denotes non-functionalised PMO containing ethylene-bridged silane units.

A considerable amount of residual hydrogen was generated when [Ru-PS] was employed as photosensitiser. Thereby, this hydrogen generation is extrinsic of CoN_5-PMO which suggested a superior selectivity in [Ru-PS]/TEOA system compared to that reported in the manuscript in Table 1 (entries 2 and 6) of 81 and 91% in the absence and the presence of BIH, respectively.

4. References

(1) Rojas-Luna, R.; Castillo-Rodríguez, M.; Ruiz, J. R.; Jiménez-Sanchidrián, C.; Esquivel, D.; Romero-Salguero, F. J. Ru- and Ir-Complex Decorated Periodic Mesoporous Organosilicas as Sensitizers for Artificial Photosynthesis. *Dalton Trans.* **2022**, *51* (48), 18708–18721. https://doi.org/10.1039/D2DT03147G.

(2) Xu, D.; Sun, Q.; Quan, Z.; Wang, X.; Sun, W. Cobalt-Catalyzed Dimerization and Homocoupling of Terminal Alkynes. *Asian J. Org. Chem.* **2018**, *7* (1), 155–159. https://doi.org/10.1002/ajoc.201700587.

(3) Singh, K.; Vellakkaran, M.; Banerjee, D. A Nitrogen-Ligated Nickel-Catalyst Enables Selective Intermolecular Cyclisation of β- and γ-Amino Alcohols with Ketones: Access to Five and Six-Membered N-Heterocycles. *Green Chem.* **2018**, *20* (10), 2250–2256. https://doi.org/10.1039/C8GC00318A.

(4) Krautwald, S.; Bezdek, M. J.; Chirik, P. J. Cobalt-Catalyzed 1,1-Diboration of Terminal Alkynes: Scope, Mechanism, and Synthetic Applications. *J. Am. Chem. Soc.* **2017**, *139* (10), 3868–3875. https://doi.org/10.1021/jacs.7b00445.

(5) Bakkali, H.; Marie, C.; Ly, A.; Thobie-Gautier, C.; Graton, J.; Pipelier, M.; Sengmany, S.; Léonel, E.; Nédélec, J.; Evain, M.; Dubreuil, D. Functionalized 2,5-Dipyridinylpyrroles by Electrochemical Reduction of 3,6-Dipyridinylpyridazine Precursors. *Eur. J. Org. Chem.* **2008**, *2008* (12), 2156–2166. https://doi.org/10.1002/ejoc.200701115.

(6) Matsumura-Inoue, T.; Tanabe, M.; Minami, T.; Ohashi, T. A Remarkably Rapid Synthesis of Ruthenium(II) Polypyridine Complexes by Microwave Irradiation. *Chem. Lett.* **1994**, *23* (12), 2443–2446. https://doi.org/10.1246/cl.1994.2443.

(7) Rosas-Hernández, A.; Steinlechner, C.; Junge, H.; Beller, M. Earth-Abundant Photocatalytic Systems for the Visible-Light-Driven Reduction of CO_2 to CO. *Green Chem.* **2017**, *19* (10), 2356–2360. https://doi.org/10.1039/C6GC03527B.

(8) Luo, J.; Zhang, J. Donor–Acceptor Fluorophores for Visible-Light-Promoted Organic Synthesis: Photoredox/Ni Dual Catalytic C(Sp^3)–C(Sp^2) Cross-Coupling. *ACS Catal.* **2016**, *6* (2), 873–877. https://doi.org/10.1021/acscatal.5b02204.

(9) Burleigh, M. C.; Markowitz, M. A.; Jayasundera, S.; Spector, M. S.; Thomas, C. W.; Gaber, B. P. Mechanical and Hydrothermal Stabilities of Aged Periodic Mesoporous Organosilicas. *J. Phys. Chem. B* **2003**, *107* (46), 12628–12634.

https://doi.org/10.1021/JP035189Q.

(10) Burleigh, M. C.; Jayasundera, S.; Thomas, C. W.; Spector, M. S.; Markowitz, M. A.; Gaber, B. P. A Versatile Synthetic Approach to Periodic Mesoporous Organosilicas. *Colloid Polym. Sci.* **2004**, *282* (7), 728–733. https://doi.org/10.1007/s00396-003-1004-0.

(11) Al-Nu'Airat, J.; Dlugogorski, B. Z.; Gao, X.; Zeinali, N.; Skut, J.; Westmoreland, P. R.; Oluwoye, I.; Altarawneh, M. Reaction of Phenol with Singlet Oxygen. *Phys. Chem. Chem. Phys.* **2018**, *21* (1), 171–183. https://doi.org/10.1039/C8CP04852E.

(12) Angeles Navarro, M.; Sain, S.; Wünschek, M.; Pichler, C. M.; Romero-Salguero, F. J.; Esquivel, D.; Roy, S. Solar Driven CO_2 Reduction with a Molecularly Engineered Periodic Mesoporous Organosilica Containing Cobalt Phthalocyanine. *Nanoscale* **2023**, *15* (5), 2114–2121. https://doi.org/10.1039/D2NR06026D.
